# Oxygenated N‑Confused Porphyrins: Structural Elucidation and Distinct Vanadium(IV) Coordination Motifs

**DOI:** 10.1021/acs.inorgchem.6c01709

**Published:** 2026-06-18

**Authors:** Bhakyaraj Kasi, Tanmoy Pain, Kai-Chun Hsu, Jian-Hong Liao, Chen-Hsiung Hung

**Affiliations:** † Institute of Chemistry, 38017 Academia Sinica, Nangang, Taipei 115201, Taiwan; ‡ Department of Chemistry, National Tsing Hua University, Hsinchu 300044, Taiwan; § Molecular Science and Technology Program, Taiwan International Graduate Program, Academia Sinica, Taipei 115201, Taiwan; ∥ Nano Science and Technology Program, Taiwan International Graduate Program, Academia Sinica, Taipei 115201, Taiwan; ⊥ Department of Chemistry, National Taiwan University, Taipei 106319, Taiwan; # Department of Chemistry, National Taiwan Normal University, Taipei 116059, Taiwan; ∇ Department of Medicinal and Applied Chemistry, Kaohsiung Medical University, Kaohsiung 807378, Taiwan

## Abstract

N-Confused porphyrinoids exhibit unusual structural and electronic properties arising from pyrrole inversion, yet the controlled oxygenation of these macrocycles remains underdeveloped because competing pathways often generate complex mixtures. Here, we identify a stepwise oxygenation sequence associated with vanadium metalation of an N-confused porphyrin (NCP) framework, providing access to structurally defined oxygenated NCP derivatives and their vanadium complexes. The lactam-containing NCP 3-oxo-*H_2_* PFNCP (**3**) was isolated as a minor coproduct in Lindsey’s N-confused porphyrin synthesis protocol using CF_3_SO_3_H as the acid catalyst. Subsequent reaction with vanadyl acetylacetonate afforded the free-base 3,21-dioxygenated NCP 3,21-dioxo-*H_3_* PFNCP (**4**) together with two distinct vanadium­(IV) complexes, V­(=O)­(OH)­(3-oxo-PFNCP) (**5**) and [*n*-Bu_4_N]­[V­(OH)­(3,21-dioxo-PFNCP)]^−^ (**6**). Single-crystal X-ray diffraction established that **6** adopts an unusual intramolecular V–O–C chelation motif arising directly from macrocycle oxygenation. UV–vis, NMR, HRMS, EPR, and electrochemical studies further revealed clear differences between **5** and **6** in their electronic structures and coordination environments. Both complexes mediate hydrogen peroxide-driven oxidation of cyclohexene and thioanisole, with **6** giving higher yields of the quantified cyclohexene oxidation products and higher selectivity for sulfone formation in thioanisole oxidation. These results demonstrate that controlled oxygenation of N-confused porphyrins can generate unusual vanadium coordination motifs with differentiated oxygen-transfer reactivity.

## Graphical abstract

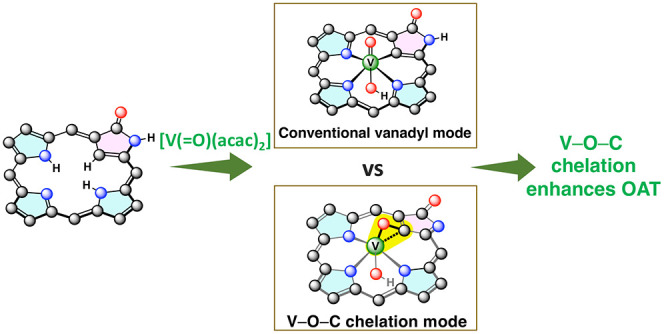

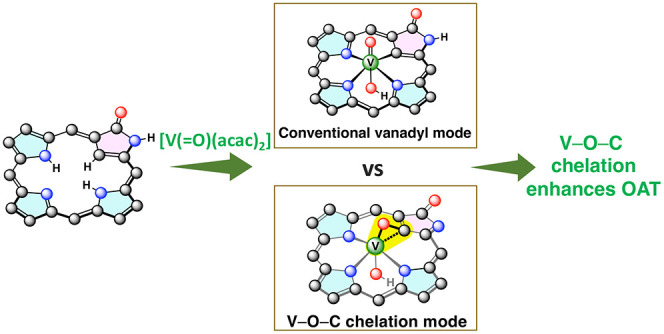

## 1 Introduction

Porphyrinoid macrocycles have long served as molecular platforms for probing structure–function relationships in molecular recognition,^1,2^ bioinspired reactivity,^3–5^ and homogeneous catalysis.^6,7^ Interest in these systems has expanded with the development of noncanonical porphyrinoid architectures, which deviate from the classical porphyrin framework and thereby access electronic structures and coordination modes unavailable to traditional porphyrins.^8^ Among them, N-confused porphyrins (NCPs)^9,10^ are particularly distinctive because inversion of one pyrrole unit reshapes both the electronic structure of the macrocycle and its intrinsic chemical reactivity.^11^

The defining topological feature of NCPs is the replacement of the conventional α,α-pyrrolic linkage by an α,β-connection, which inverts one pyrrole ring and exposes unusual reactive sites within the macrocyclic core.^12^ This “confusion” enables bonding motifs rarely encountered in canonical porphyrins, most notably metal–carbon interactions, while also opening direct functionalization pathways at noncanonical positions.^11^ As a result, NCPs provide a platform for exploring unconventional coordination chemistry,^13^ organometallic-type reactivity,^14^ and site-selective macrocycle modification.^15^ Systematic derivatization at the outer nitrogen (2N),^16^ the *β*-carbon of the inverted pyrrole (C3),^17,18^ and the inner carbon (C21)^19^ has shown that subtle changes in connectivity can strongly influence redox properties, metal binding, and catalytic behavior.^20^

Within this context, oxygenated NCP derivatives are intriguing. Introduction of carbonyl or related oxygen functionalities can perturb aromatic delocalization while simultaneously installing harder donor sites capable of secondary coordination, hydrogen bonding, or metal–ligand cooperativity.^21,22^ Such modifications are expected to affect both thermodynamic preferences, including metal-ion affinity and coordination environment, and kinetic features relevant to O–O bond activation and oxygen atom transfer. Early studies indeed suggested that oxo-functionalization can stabilize unusual oxidation states and unlock reactivity patterns distinct from those of nonoxygenated NCPs.^23,24^ Nevertheless, selective oxygenation of NCP-derived porphyrins to yield isolable compounds remains uncommon. Oxygenated species are often obtained only as minor byproducts or transient intermediates, and unambiguous structural characterization is frequently challenging.^25^ As a result, the influence of oxygenation on the coordination geometry and reactivity remains insufficiently understood.

These issues are particularly important for oxidation catalysis by the NCP metal complexes. Oxygen atom transfer (OAT) and related peroxide-driven oxidations are fundamental to many biological and synthetic processes.^26,27^ Enzymatic systems such as heme-dependent monooxygenases and peroxidases achieve high selectivity through precise control of the macrocyclic framework and secondary coordination sphere.^28,29^ Although synthetic metalloporphyrins have provided important biomimetic models,^30^ comparable control in noncanonical porphyrinoids, especially oxygenated derivatives, remains limited. Vanadium is relevant in this regard because it can adopt multiple oxidation states and coordination numbers, and vanadium-dependent haloperoxidases provide precedents for peroxide-driven oxidation chemistry.^31^ Incorporating vanadium into an oxygenated NCP or N-confused porphyrin framework, therefore, offers an opportunity to modulate metal-centered reactivity through macrocycle-level electronic and structural perturbation.

Here, we address this synthetic challenge by identifying a stepwise oxygenation sequence during vanadium metalation of an N-confused porphyrin (NCP) framework as shown in Chart [Fig cht1-fo]. We isolated the free-base lactam-containing NCP, 3-oxo-*H*_2_PFNCP (**3**), as a minor coproduct in Lindsey’s N-confused porphyrin synthesis protocol using CF_3_SO_3_H as the acid catalyst, and found that subsequent vanadium insertion afforded two vanadium­(IV) NCP complexes, V­(=O)­(OH)­(3-oxo-PFNCP) (**5**) and [*n*-Bu_4_N]^+^[V­(OH)­(3,21-dioxo-PFNCP)]^−^ (**6**), together with the free-base 3,21-dioxygenated NCP, 3,21-dioxo-*H*_3_PFNCP (**4**), whose structure was established by single-crystal X-ray diffraction. Despite sharing the same molecular formula, **5** and **6** display distinct oxygenation and coordination motifs, including an unusual intramolecular V–O–C interaction in **6**. These structural differences are accompanied by divergent reactivity in H_2_O_2_-driven cyclohexene and thioanisole oxidation. Overall, this study demonstrates that controlled NCP oxygenation and vanadium coordination can cooperatively generate unusual vanadium coordination environments with differentiated oxygen-transfer reactivity.

**1 cht1-fo:**
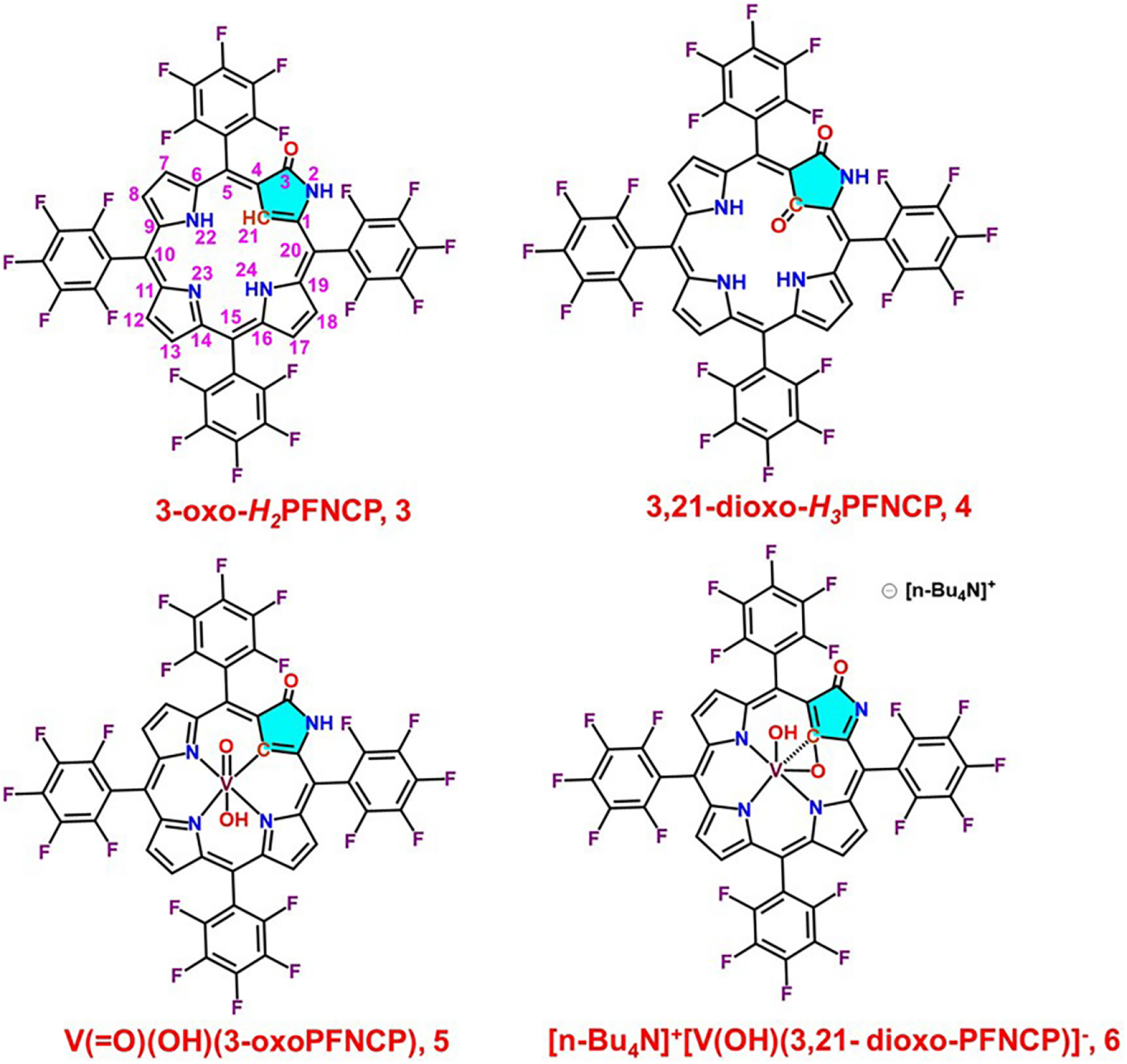
Molecular structures of 3-oxo-*H_2_* PFNCP (**3**), 3,21-dioxo-*H_3_* PFNCP (**4**), [V­(=O)­(OH)­(3-oxo-PFNCP)] (**5**), and [*n*-Bu_4_N]^+^ [V­(OH)­(3,21-dioxo-PFNCP)]^−^ (**6**). PFNCP denotes the tetrakis­(pentafluorophenyl)-N-confused porphyrin ligand. The NCP atom-numbering scheme is shown for structure **3**.

## 2 Results and Discussion

### 2.1 Synthesis and Isolation of Oxygenated NCPs and Their Vanadium Complexes

The free-base pentafluorophenyl N-confused 3-oxoporphyrin (3-oxo-*H*_2_PFNCP, **3**) was isolated as a minor oxygenated coproduct from Lindsey’s one-pot, two-step N-confused porphyrin synthesis protocol using CF_3_SO_3_H as the acid catalyst (Scheme 1). Under these conditions, **3** likely arises from *in situ* oxygenation at the C3 position of the inverted pyrrole and was isolated in 4.2% yield by chromatography after separation from pentafluorophenylporphyrin (*H*_2_TPFPP, **1**, 39%) and tetrakis­(pentafluorophenyl)-N-confused porphyrin (*H*_2_PFNCP, **2**, 8.5%). This route contrasts with previously reported approaches to 3-oxo N-confused porphyrins, which typically rely on multistep sequences involving functionalization of metalated intermediates followed by hydrolysis and demetalation.^23^

**Scheme 1:**
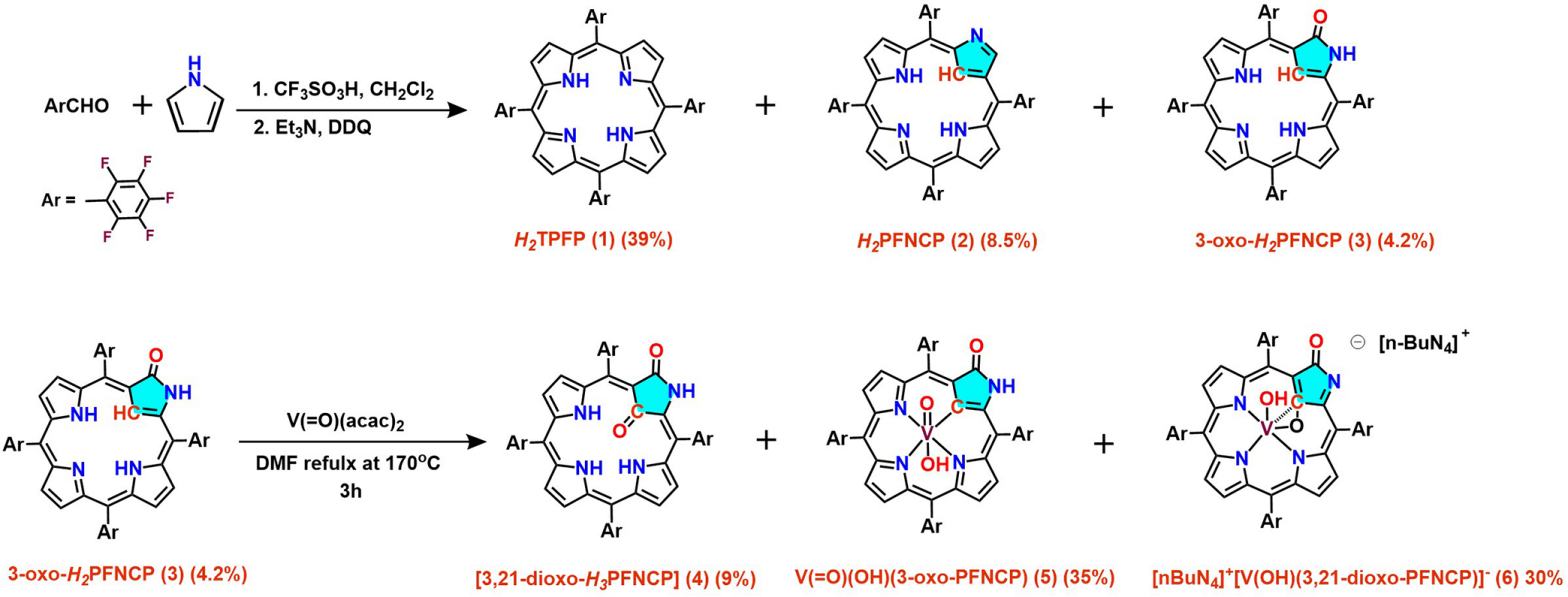
Synthetic Routes and Isolated Yields for 3-oxo-*H*_*2*_PFNCP (**3**), 3,21-dioxo-*H*_*3*_PFNCP (**4**), V­(=O)­(OH)­(3-oxo-PFNCP) (**5**), and [*n*-Bu_4_N]^+^[V­(OH)­(3,21-dioxo-PFNCP)]^−^ (**6**)

Metalation of **3** with vanadyl acetylacetonate [V­(=O)­(acac)_2_] in refluxing DMF afforded a mixture of oxygenated products, from which three discrete compounds were isolated: the free-base 3,21-dioxygenated N-confused porphyrin (3,21-dioxo-*H*_3_PFNCP, **4**, 9%) and two structurally distinct vanadium­(IV) complexes, **5** (35%) and **6** (30%). Complex **5** corresponds to a vanadyl N-confused 3-oxoporphyrin bearing an axial hydroxide ligand trans to the terminal V=O group, consistent with a conventional vanadyl coordination environment. By contrast, complex **6** is a structurally unusual vanadium­(IV) derivative in which the oxygenated macrocycle coordinates directly through an intramolecular V–O–C chelation motif involving an alkoxide-type oxygen donor, together with an additional hydroxide ligand. The concomitant formation of free-base **4** is most plausibly attributed to partial demetalation of a 3,21-dioxygenated vanadium intermediate related to **6**, since both compounds share the same oxygenation pattern on the NCP framework.

All isolated compounds were characterized by elemental analysis, ESI-MS, UV–vis, FT-IR, and, where appropriate, NMR or EPR spectroscopy. Definitive structural assignments were established by single-crystal X-ray diffraction, which confirmed the molecular identities, oxygenation patterns, and coordination modes of the isolated compounds (Figure 1 and Table S3).

**Figure 1:**
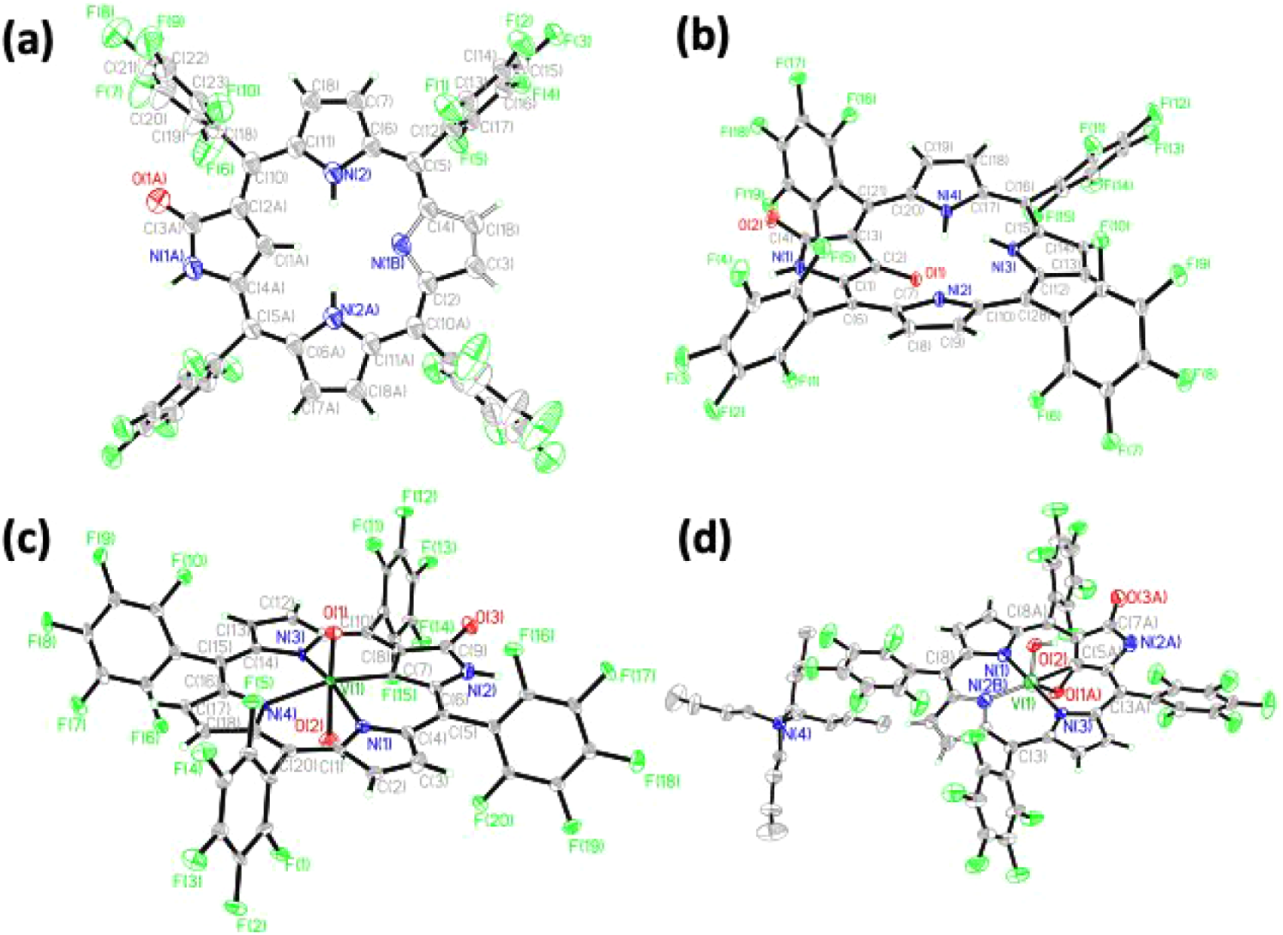
Single-crystal X-ray structures of the free-base compounds (a) **3** and (b) **4**, and the vanadium complexes (c) **5** and (d) **6**.

## 3 Solid-State Structures and Coordination Consequences of Oxygenation

Single-crystal X-ray diffraction analyses of **3**–**6** reveal how stepwise oxygenation reshapes the macrocyclic geometry and, in the vanadium complexes, gives rise to two distinct coordination motifs (Figure 1). Although all four structures exhibit some degree of positional disorder, arising from the small electron-density differences between carbon and nitrogen atoms in the inverted pyrrole unit and the absence of a strongly preferred orientation during metalation, the crystallographic data are sufficient for meaningful structural comparison.

The free-base N-confused 3-oxoporphyrin **3** crystallizes in the trigonal space group *R*3̅(H), with one-half of the molecule in the asymmetric unit and the remainder generated by crystallographic symmetry. The lactam-containing inverted pyrrole ring is disordered over two symmetry-related positions associated with an inversion center. Despite this disorder, the macrocycle remains essentially planar, with a mean deviation of 0.044 Å across the 25 core atoms. The lactam unit is clearly established by the C­(3A)–O­(1A) and C­(3A)–N­(1A) bond lengths of 1.278(13) and 1.331(11) Å, respectively, consistent with substantial amide character. The pentafluorophenyl substituents are oriented nearly orthogonal to the macrocyclic plane, with dihedral angles of 81.32° and 78.77°, thereby minimizing steric congestion and π-π interactions.

In contrast, the single-crystal structure of 3,21-dioxo-*H*_3_PFNCP (**4**) reveals a substantially more distorted macrocyclic framework. Compound **4** crystallizes in the triclinic space group *P*1̅ and contains two crystallographically independent molecules in the asymmetric unit. In both molecules, the outer C(4) carbon and the peripheral nitrogen atom of the inverted pyrrole show 50% occupational disorder, giving rise to two alternative orientations of the lactam carbonyl groups. Unlike the nearly planar structure of **3**, compound **4** adopts a highly nonplanar conformation, with a mean deviation of 0.238 Å across the 26 core atoms. The combination of three pyrrolic N–H protons and an inner carbonyl group creates a congested central cavity, and intramolecular hydrogen bonding between the inner carbonyl oxygen and adjacent pyrrolic N–H groups pulls three pyrrole units toward one face of the macrocycle while the remaining pyrrole tilts in the opposite direction, producing pronounced saddle-type distortion. The pyrrolidine-2,4-dione moiety is clearly resolved, with C(2)–O(1) and C(4)–O(2) bond lengths of 1.290(4) and 1.265(6) Å, respectively. The adjacent C–C single bonds [C(1)–C(2) = 1.430(5) Å; C(2)–C(3) = 1.440(5) Å] further support partial localization of the π-system and diminished global aromaticity.

The vanadium complex V­(=O)­(OH)­(3-oxo-PFNCP) (**5**) retains a largely planar N-confused porphyrinoid core closely resembling that of its free-base precursor **3**. The lactam C=O bond length [C(9)–O(3) = 1.233(12) Å] is comparable to that in **3**, indicating that metalation does not substantially perturb this portion of the macrocycle. Asymmetric axial coordination displaces the vanadium atom by 0.317 Å from the mean porphyrin plane toward the terminal oxo ligand. At the metal center, the axial oxo and hydroxo ligands are disordered over two positions with a refined occupancy ratio of 60:40. Despite this disorder, the refined V(1)=O(1) distance of 1.552(4) Å is consistent with a vanadyl­(IV) center. In contrast, the apparent V–OH distance [V(1)–O(2) = 2.253­(4) Å] is substantially longer than typical V–OH bond lengths of 1.85–1.91 Å and most likely reflects positional disorder of the axial ligands.^32^ The short V(1)=O(1) distance also agrees well with those reported for related vanadium­(IV) porphyrinoid complexes, further supporting the assigned coordination environment.^33^

A different coordination mode is observed for [*n*-Bu_4_N]^+^[V­(OH)­(3,21-dioxo-PFNCP)]^−^ (**6**), which crystallizes as a tetra-*n*-butylammonium salt in the orthorhombic space group *C*222_1_, with one-half of the anionic complex and one-half of the counterion in the asymmetric unit. In contrast to **5**, the porphyrinoid core of **6** is nearly planar, with a mean deviation of only 0.027 Å across the 25 porphyrinic atoms, and the vanadium atom lies close to the macrocyclic center. The absence of a terminal axial oxo ligand is supported by the observed V–OH bond distance [V(1)–O(2) = 1.898(14) Å], while the peripheral carbonyl C­(7A)=O­(3A) bond length of 1.207(12) Å supports the assignment of a lactam-functionalized macrocycle. Most notably, the inverted pyrrole ring adopts a deprotonated 4-hydroxypyrrol-2-one-like motif, enabling intramolecular coordination of the inner-carbon oxygen atom to the vanadium center. The inner C­(5A)–O­(1A) bond length of 1.41(2) Å is consistent with predominant single-bond character, whereas the V(1)–O­(1A) distance of 1.752(15) Å indicates a substantial metal–oxygen interaction. In addition, the close V(1)–C­(5A) separation of 2.146(14) Å suggests an additional metal–carbon interaction, consistent with the DFT-optimized geometries (Figure 2, Figures S19–S26 and Table S3). Taken together, these structural parameters establish **6** as a rare vanadium­(IV) N-confused oxoporphyrin featuring an intramolecular V–O–C chelation motif arising directly from macrocycle oxygenation.

**Figure 2:**
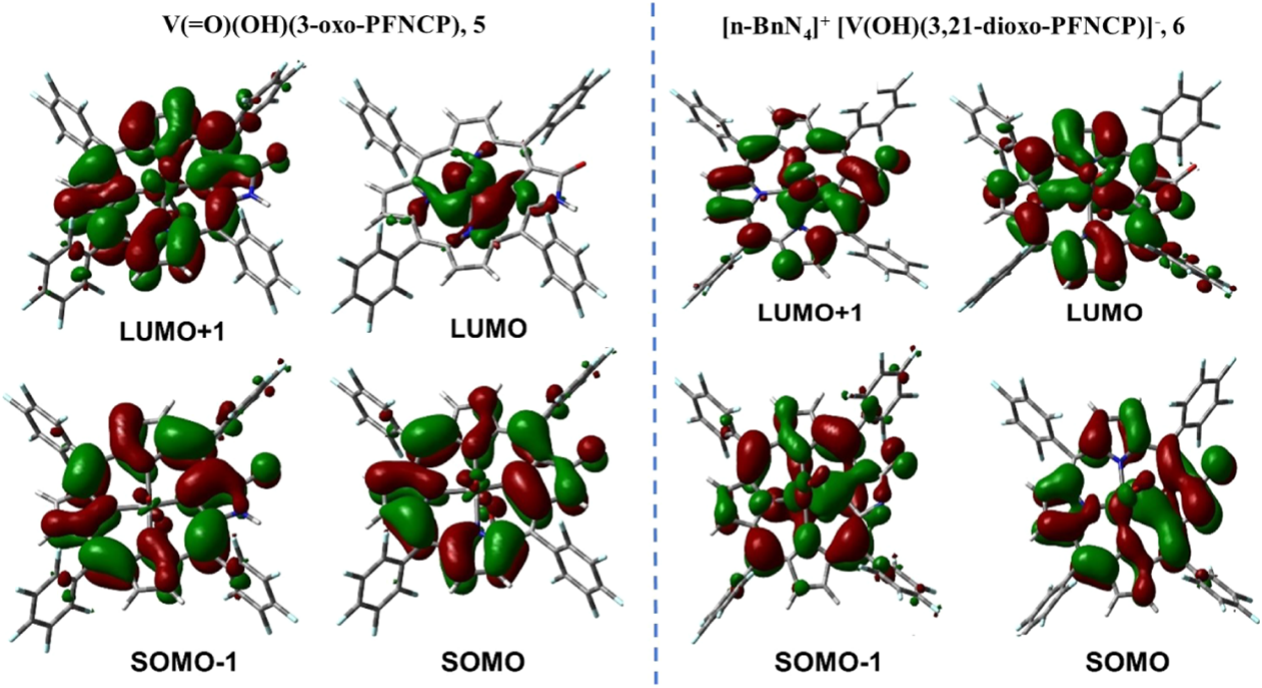
Selected frontier molecular orbitals of compounds **5** and **6**.

## 4 Solution-Phase Spectroscopic and Electronic Features

### 4.1 UV–Vis and NMR/HRMS Characterization

Electronic absorption spectroscopy further supports the structural differentiation among **3–6** (Figure 3 and Table S2). The spectra of the vanadium complexes broadly resemble those of their corresponding free-base ligands, indicating that the porphyrinoid π-system remains the principal determinant of the optical response after metalation. Free-base **3** shows a characteristic porphyrinoid profile with an intense Soret band at 433 nm (ε = 1.70 × 10^5^ M^–1^ cm^–1^) and four well-resolved Q bands over 500–700 nm, consistent with a largely delocalized 18π-aromatic NCP framework. In contrast, **4** displays marked attenuation and broadening of the high-energy transition, with a split Soret band and poorly resolved Q bands, consistent with conversion of the inverted pyrrole segment into a pyrrolidinedione-like motif that disrupts conjugation and substantially diminishes aromatic character.^34^

**Figure 3:**
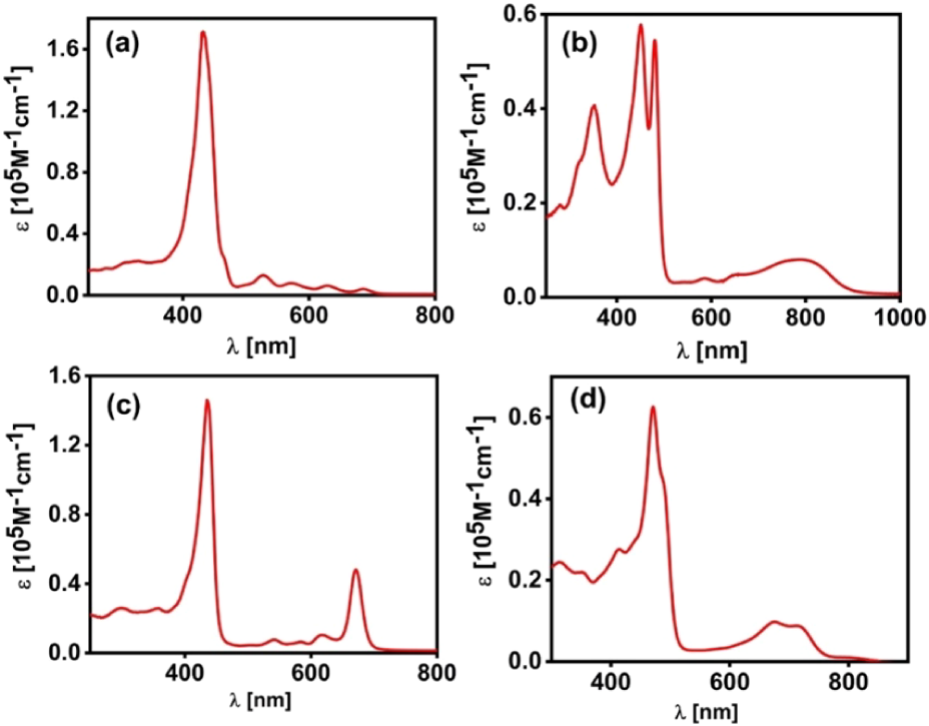
Electronic absorption spectra of (a) 3-oxo-*H*_2_PFNCP (**3**); (b) 3,21-dioxo-*H*_3_PFNCP (**4**); (c) V­(=O)­(OH)­(3-oxo-PFNCP) (**5**); and (d) [*n*-Bu_4_N]^+^[V­(OH)­(3,21-dioxo-PFNCP)]^−^ (**6**), all in CH_3_CN.

Metalation of **3** to give **5** largely preserves the porphyrinoid absorption pattern, although the Soret band becomes modestly less intense and the lowest-energy Q-band region near 672 nm gains intensity, consistent with metalation-induced symmetry lowering and macrocycle distortion. By contrast, **6** exhibits a substantially red-shifted and attenuated Soret band at 471 nm together with a broad absorption envelope spanning 600–800 nm, reflecting both reduced delocalization of the oxygenated NCP scaffold and pronounced electronic perturbation associated with the intramolecular V–O–C chelation motif.

NMR spectroscopy similarly distinguishes the two oxygenated free-base ligands. For **3**, the ^1^H NMR spectrum shows six β-pyrrolic resonances in the aromatic region at δ 8.82–8.56 ppm, a lactam N–H resonance at δ 8.85 ppm, two inner N–H protons at δ −2.80 ppm, and an inner C–H resonance at δ −5.47 ppm. These assignments are supported by two-dimensional COSY and HSQC data. By contrast, **4** exhibits seven β-pyrrolic signals between δ 8.05 and 7.45 ppm and a peripheral N–H singlet at δ 7.69 ppm, consistent with a weakly aromatic and perturbed π-system with partial ring current effects.^35,36^ The three inner N–H protons appear in two distinct environments, with two signals at δ 6.92–6.89 ppm and a third at δ −0.35 ppm, consistent with an asymmetric hydrogen-bonding environment involving the inner carbonyl oxygen. The two downfield N–H signals are assigned to protons more strongly affected by N–H···O hydrogen bonding, whereas the moderately upfield resonance at δ −0.35 ppm is attributed to a less hydrogen-bonded N–H proton located in a different local shielding environment within the distorted macrocyclic cavity. As expected for V­(IV) centers, **5** and **6** show broadened and unresolved ^1^H NMR signals and are not amenable to detailed NMR assignment.

High-resolution ESI mass spectrometry supports the molecular formulations. Compound **3** exhibits a deprotonated ion at *m*/*z* 989.0 ([M–H]^−^), compound **4** shows a molecular ion peak at *m*/*z* 1006.0 ([M]^+^), and complexes **5** and **6** give peaks at *m*/*z* 1069.9626 and 1069.9629, respectively. Although **5** and **6** share the same nominal composition, their distinct spectroscopic and crystallographic behavior clearly establish that they represent different oxygenation and coordination isomers rather than simply species of the same composition by MS.

### 4.2 EPR Studies

X-band EPR spectroscopy provides a secure assignment of the metal oxidation state and overall electronic configuration in the vanadium complexes (Figure 4 and Table 1). Both **5** and **6** display well-resolved axial spectra at 77 K with the characteristic eight-line hyperfine splitting pattern expected for coupling of the unpaired electron to the ^51^V nucleus (I = 7/2), thereby confirming V­(IV) (d^1^) electronic structures in both complexes.

**Figure 4:**
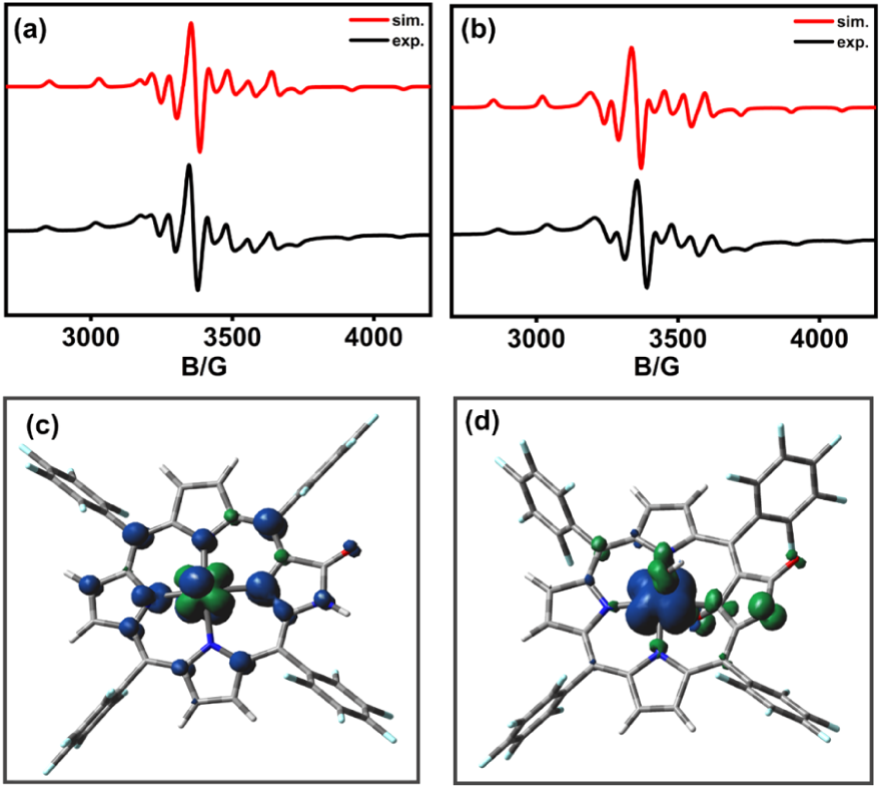
Solid-state X-band EPR spectra of compounds **5** and **6** measured at 77 K: (a) EPR spectrum of **5** and (b) EPR spectrum of **6**, with the red traces corresponding to simulated spectra and the black traces corresponding to experimental spectra. Spin-density plots of (c) compound **5** and (d) compound **6**.

**Table 1:** Spin Hamiltonian Parameters from Experimental and Simulated EPR Spectra of Compounds **5** and **6**

	Experimental				Simulated			
Compound	*g*_⊥_	*g*_∥_	*A*_⊥_ (×10^–4^ cm^–1^)	*A*_∥_ (×10^–4^ cm^–1^)	*g*_⊥_	*g*_∥_	*A*_⊥_ (×10^–4^ cm^–1^)	*A*_∥_ (×10^–4^ cm^–1^)
**5**	1.988	1.957	60.6	163.4	1.986	1.950	61.4	163.5
**6**	1.980	1.950	63.7	160.6	1.989	1.957	64.7	161.4

The extracted spin Hamiltonian parameters are consistent with axially anisotropic vanadyl-type environments, with *g*_⊥_ > *g*_∥_ and *A*_∥_ ≫ *A*_⊥_. The modest differences in *g* and *A* values between **5** and **6** are consistent with their distinct coordination environments, while the absence of well-resolved superhyperfine coupling to pyrrolic nitrogens suggests that the unpaired electron remains largely localized on vanadium in both cases. Consistent with this interpretation, the calculated SOMO and spin-density distributions indicate predominantly V-centered unpaired-electron character, while **6** shows more evident extension toward the V–O–C chelate region, reflecting enhanced electronic coupling between vanadium and the oxygenated inverted-pyrrole fragment. In the present study, the EPR data primarily verify that both complexes are V­(IV) derivatives despite their markedly different coordination motifs.

### 4.3 Infrared and Electrochemical Comparison

FT-IR spectroscopy provides supporting evidence for the oxygenation patterns and coordination motifs assigned above. All compounds show N–H stretching bands in the 3373–3290 cm^–1^ range together with carbonyl absorptions between 1733 and 1695 cm^–1^, consistent with lactam and dione functionalities.^37–39^ In particular, **5** shows a characteristic V=O stretch at 951 cm^–1^, as expected for a terminal vanadyl unit, whereas **6** displays additional bands at 487 and 1324 cm^–1^ assignable to V–O and C–O modes associated with intramolecular oxygen coordination.^40,41^ These assignments are further supported by the DFT-calculated vibrational features, which are consistent with the diagnostic V=O mode for **5** and the V–O/C–O modes associated with the V–O–C chelation motif in **6**.

Cyclic voltammetry further differentiates complexes **5** and **6**, although the assignments of the individual redox processes were not further verified by spectroelectrochemical methods (Figure 5 and Table 2). Complex **5** displays two reversible reduction waves at −0.48 and −0.88 V that are absent in the voltammogram of free-base **3**, consistent with metal-centered redox activity. Complex **6**, by contrast, shows a distinct oxidation process at 0.33 V, tentatively assigned as ligand-based and plausibly associated with the more electron-rich environment created by intramolecular V–O–C chelation. At higher potentials, both complexes exhibit a sharp increase in the anodic current. These irreversible features may reflect oxidative ligand degradation, polymerization, or electrocatalytic oxidation of residual water; however, further mechanistic studies are required to definitively assign these events.

**Figure 5:**
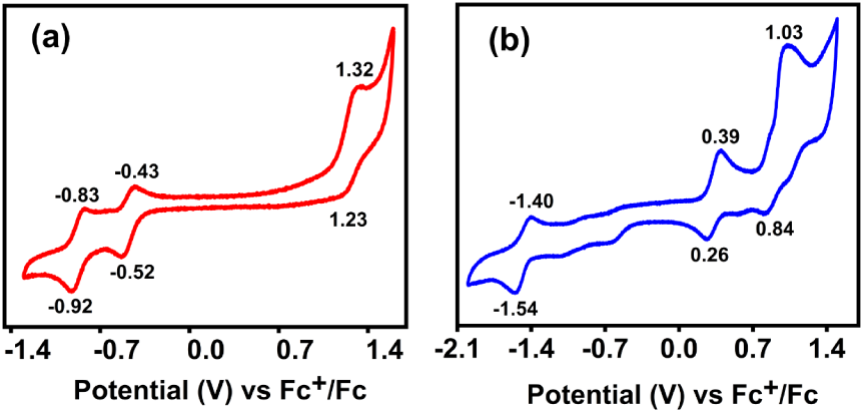
Cyclic voltammograms of (a) compound **5** and (b) compound **6** recorded in CH_2_Cl_2_, with potentials referenced to the Fc^0^/Fc^+^ couple.

**Table 2:** Electrochemical Data of the Synthesized Compounds **5** and **6**

	Potential (V vs Fc/Fc^+^)			
Compounds	Red 2	Red 1	Ox 1	Ox 2
5	–0.88	–0.48		1.32^a^
6	–1.47		0.33	1.03^a^

a Anodic Peak Potential.

## 5 Comparative Oxygen-Transfer Reactivity of **5** and **6**

The catalytic oxygenation performance of the synthesized vanadium­(IV) complexes was evaluated using cyclohexene and thioanisole as representative substrates and hydrogen peroxide (30 wt %) as an environmentally benign terminal oxidant. Under these conditions, cyclohexene oxygenation afforded four major products—cyclohexene oxide, 2-cyclohexen-1-ol, 2-cyclohexen-1-one, and cyclohexane-1,2-diol—whereas thioanisole oxygenation produced the corresponding sulfoxide and sulfone (Schemes 2 and 3).

**Scheme 2:**
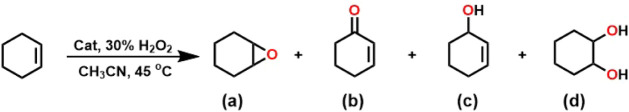
Catalytic Oxidation of Cyclohexene with Vanadium Complexes Using H_2_O_2_ as Oxidant

**Scheme 3:**
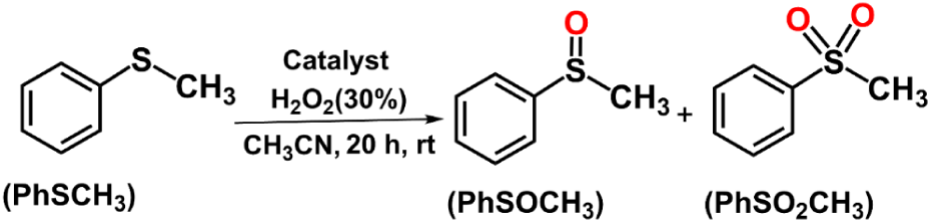
Catalytic Oxidation of Thioanisole by Vanadium­(IV) Complexes Using H_2_O_2_ as Oxidant

### 5.1 Cyclohexene Oxidation

Optimization experiments using **6** indicate that the catalyst loading, H_2_O_2_ concentration, substrate concentration, and temperature all affect the reaction outcome (Table 3). However, two aspects of the data set require cautious interpretation. First, cyclohexene conversion often exceeds the combined yield of the four quantified products by a substantial margin, indicating that a significant fraction of the reacted substrate is converted into unidentified products, possibly including overoxidized or higher molecular weight species. Second, the dependence of the combined product yield on the H_2_O_2_ concentration is nonmonotonic, with higher peroxide loadings giving lower combined yields under otherwise similar conditions. Collectively, these observations suggest that excess H_2_O_2_ may promote unproductive decomposition pathways, nonselective substrate oxidation, and/or catalyst deactivation, thereby precluding rigorous rate law analysis under the present conditions.

**Table 3:** Cyclohexene Oxidation under Various Reaction Conditions Using **6** as the Catalyst^a^

							Product Distribution (%)^d^				
No.	[Cat] (M)	[Subst.] (M)	[H_2_O_2_] (M)	Temp (°C)	Subst. Consumption (%)^b^	Combined Yield (%)^c^	Oxide (a)	Keto (b)	OH (c)	Diol (d)	TOF (h^–1^)
1	0	0.125	0.2	45	35	0	0	0	0	0	0
2	0.001	0.125	0	45	44	0	0	0	0	0	0
3	0.0001	0.125	0.2	45	72	7.7	0	60	40	0	4.04
4	0.001	0.125	0.2	45	98	8.5	34	33	16	14	0.44
5	0.001	0.125	0.3	45	92	23	13	41	33	13	1.2
6	0.002	0.125	0.2	45	79	38	12	35	33	20	1.09
7	0.002	0.125	0.3	45	93	9	0	49	29	22	0.25
8	0.002	0.125	0.4	45	98	12	4	53	21	22	0.31
9	0.002	0.2	0.2	45	65	42	10	46	22	22	1.10
10	0.002	0.125	0.2	60	100	30	0	41	18	41	0.77

a General reaction conditions: reaction time, 24 h; solvent, CH_3_CN; and oxidant, 30% H_2_O_2_.

b Substrate consumption (%) = [(initial moles of cyclohexene – remaining moles of cyclohexene)/initial moles of cyclohexene] × 100.

c The combined yield (%) was calculated as (total moles of the four quantified products/initial moles of cyclohexene) × 100.

d Product distribution (%) = (moles of an individual product/total moles of the four quantified products) × 100.

Under the conditions that afforded the highest combined yield of the four quantified products—complex **6** (0.002 M), cyclohexene (0.125 M), and H_2_O_2_ (0.2 M) in CH_3_CN at 45 °C for 24 h—cyclohexene conversion reached 79%, while the combined yield of cyclohexene oxide (a), 2-cyclohexen-1-one (b), 2-cyclohexen-1-ol (c), and cyclohexane-1,2-diol (d) was 38%. The quantified products were distributed as 12% epoxide (a), 35% allylic ketone (b), 33% allylic alcohol (c), and 20% diol (d), indicating that both epoxidation and allylic oxidation pathways contribute to the observed reactivity. Because a substantial fraction of the converted cyclohexene is not accounted for by these four products, these data are interpreted cautiously as reflecting comparative reactivity rather than a complete catalytic profile.

The direct comparison between **5** and **6** provides a clearer assessment than the optimization data set alone (Table 4 and Figure 6). Although **5** shows slightly higher cyclohexene conversion than **6** (84% vs 79%), **6** afforded a higher combined yield of the four quantified products (38% vs 26%) and a higher TOF (1.09 vs 0.67 h^–1^). Thus, **6** is more effective at directing cyclohexene conversion toward the identified oxygenated products, whereas **5** gives a larger fraction of unidentified or unquantified products. The time-dependent product-formation profiles are consistent with this interpretation, showing faster accumulation of the quantified products for **6** over the course of the reaction.

**Figure 6:**
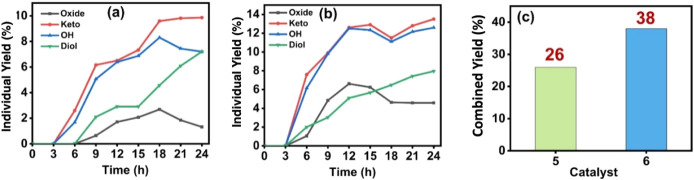
Time-dependent formation profiles of the four quantified oxidation products in cyclohexene oxidation catalyzed by compounds **5** (a) and **6** (b). (c) Comparison of the total conversion of the four quantified products and their distributions.

**Table 4:** Comparative Analysis of Cyclohexene Oxidation Catalyzed by Compounds **5** and **6** ^a^

					Product Distribution (%)^d^				
No.	Cat.	Time (h)	Subst. Consumption (%)^b^	Combined Yield (%)^c^	Oxide	Keto	OH	Diol	TOF (h^–1^)
1	**5**	24	84	26	5	39	28	28	0.67
2	**6**	24	79	38	12	35	33	20	1.09

a General conditions: catalyst (0.002 M), cyclohexene (0.125 M), and H_2_O_2_ (0.2 M) in CH_3_CN (5 mL) at 45 °C for 24 h.

b Substrate consumption (%) = [(initial moles of cyclohexene – remaining moles of cyclohexene)/initial moles of cyclohexene] × 100.

c Combined yield (%) = (total moles of the four quantified products/initial moles of cyclohexene) × 100.

d Product distribution (%) = (moles of an individual product/total moles of the four quantified products) × 100.

### 5.2 Thioanisole Oxidation

Thioanisole oxidation provides a cleaner benchmark for comparing **5** and **6** because the product manifold is limited to sulfoxide and sulfone (Table 5 and Figure 7). In the absence of a catalyst, thioanisole (0.125 M) reacts with H_2_O_2_ (0.65 M) in CH_3_CN at room temperature to give a 27% combined yield after 20 h, with exclusive formation of sulfoxide. By contrast, in the absence of H_2_O_2_, neither **5** nor **6** shows any detectable reactivity, confirming that the oxidant is indispensable, while the vanadium complexes enhance activity and alter product selectivity.

**Figure 7:**
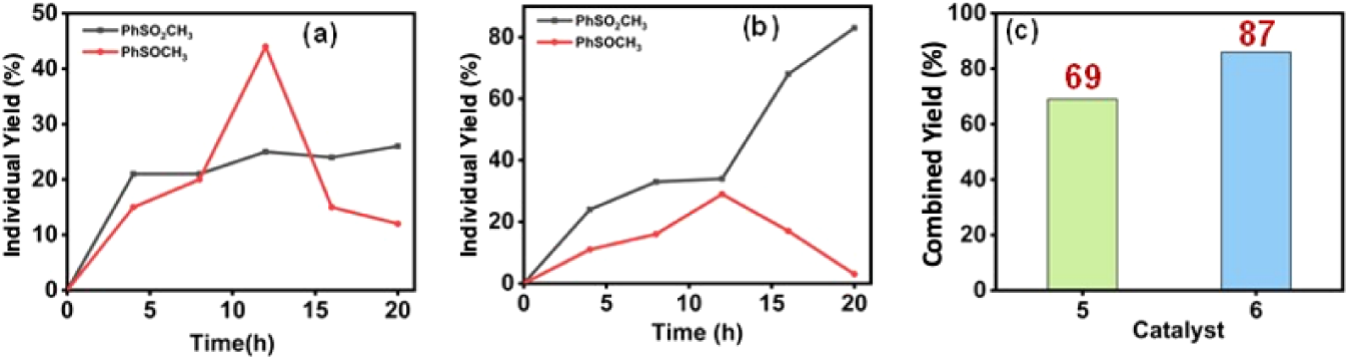
Time-dependent profiles for thioanisole oxidation catalyzed by compounds **5** (a) and **6** (b). (c) Comparison of total conversion and product selectivity for both catalysts.

**Table 5:** Comparative Catalytic Performance of Vanadium­(IV) Complexes **5** and **6** in Thioanisole Oxidation^a^

							Product Distribution (%)^d^		
No.	Cat.	. Cat.(M)	Time (h)	H_2_O_2_ (M)	Combined Yield (%)^b^	Subst. Consumption (%)^c^	PhSO_2_CH_3_	PhSOCH_3_	TOF (h^–1^)
1	-	0	20	0.65	27	27	0	100	0
2	5	0.0006	20	-	0	0	0	0	0
3	6	0.0006	20	-	0	0	0	0	0
4	5	0.0006	12	0.65	69	95	36	64	11.94
5	5	0.0006	20	0.65	38	99	67	33	3.98
6	6	0.0006	20	0.65	87	99	96	4	8.99

a General reaction conditions: thioanisole (0.125 M) in CH_3_CN at room temperature.

b The combined yield (%) was calculated as (total moles of sulfoxide and sulfone formed/initial moles of thioanisole) × 100.

c Substrate consumption (%) = [(initial moles of substrate – remaining moles of substrate)/initial moles of substrate] × 100.

d Product distribution (%) = (moles of an individual product/total moles of sulfoxide and sulfone formed) × 100.

Upon addition of H_2_O_2_, reactions containing **5** or **6** showed increased substrate conversion and greater sulfone formation relative to the background reaction in the absence of a catalyst. Under comparable conditions, **5** affords a 69% combined yield after 12 h with 95% substrate consumption, giving a mixture of sulfoxides and sulfones in a 64:36 ratio. Extending the reaction time to 20 h lowers the combined yield of sulfoxide and sulfone to 38%, with the product ratio shifting to 33:67, likely due to product decomposition and secondary side reactions under the prolonged oxidative conditions. In contrast, complex **6** affords an 87% combined yield after 20 h with 99% substrate consumption and a high sulfone fraction, giving a product distribution of 96% PhSO_2_CH_3_ and 4% PhSOCH_3_. The time-dependent profiles support a sequential oxidation scenario: for **5**, sulfoxide accumulates at early times and then decreases as sulfone forms, whereas for **6**, sulfoxide remains a minor transient intermediate and is further oxidized to a sulfone. Thus, both catalysts promote sulfur oxidation beyond the background reaction, but **6** more effectively drives the reaction toward the sulfone product.

The higher product yield obtained with **6** may be associated with its distinctive intramolecular V–O–C chelation motif, which modifies the electronic environment of the vanadium center and likely facilitates peroxide activation and subsequent oxygen atom transfer. In this sense, the sulfur oxidation data provide a clear structure–reactivity correlation in the present study, highlighting the influence of subtle metal–ligand structural variation on both catalytic efficiency and product selectivity.

### 5.3 Tentative Mechanistic Implications

The present data do not allow definitive identification of the catalytically active intermediates, but they support a cautious working model. Negative-mode ESI-MS monitoring revealed anionic signals consistent with peroxide-derived vanadium species after the treatment of **6** with H_2_O_2_, as presented in Scheme 4. Because ESI-MS selectively detects charged species, these ions may correspond to deprotonated or ionization-stabilized transient species rather than the dominant catalytic intermediates. Nevertheless, their observation suggests that H_2_O_2_ can associate with or be activated by the vanadium center.

**Scheme 4:**
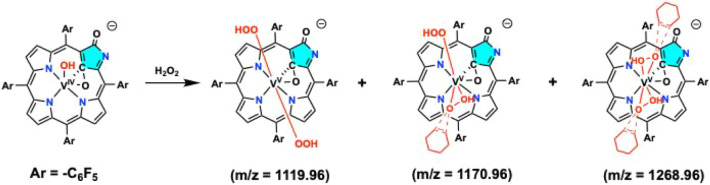
Tentative Working Model for Peroxide-Derived Anionic Vanadium Species Detected by Negative-Mode ESI-MS after Treatment of **6** with H_2_O_2_ ^a^

a The proposed structures represent plausible formulations of the detected ions rather than confirmed dominant solution species. Dashed bonds indicate possible substrate/intermediate interactions during oxygen transfer.

In this context, the intramolecular V–O–C chelation motif in **6** may influence the local electronic structure and proton-transfer environment around vanadium, thereby facilitating H_2_O_2_ binding, deprotonation, or formation of peroxide-derived intermediates. The DFT results provide qualitative support for this interpretation. Because the SOMO represents the singly occupied orbital of the V­(IV), d^1^ center, its spatial distribution reflects the unpaired-electron character. Compared with **5**, the SOMO of **6** extends more evidently over the V–O–C chelate region, indicating enhanced electronic coupling between vanadium and the oxygenated inverted-pyrrole fragment. This redistribution may help accommodate electron-density changes during peroxide activation and could contribute to oxygen-transfer or radical-involved oxygenation pathways.

Accordingly, direct participation of the intramolecular oxygen donor in proton transfer or oxygen transfer processes cannot be excluded. This interpretation is consistent with the higher combined yield of quantified oxidation products in cyclohexene oxidation and the higher sulfone fraction in thioanisole oxidation observed for **6** relative to that for **5**. However, because the proposed intermediates are inferred mainly from negative-mode ESI-MS and were not independently verified, their exact identities remain tentative.

## 6 Conclusion

In summary, we have identified a stepwise oxygenation sequence within an N-confused porphyrin framework that provides access to two oxygenated free-base macrocycles, 3-oxo-*H*_2_PFNCP (**3**) and 3,21-dioxo-*H*_3_PFNCP (**4**), together with two structurally distinct vanadium­(IV) complexes, V­(=O)­(OH)­(3-oxo-PFNCP) (**5**) and [*n*-Bu_4_N]^+^[V­(OH)­(3,21-dioxo-PFNCP)]^−^ (**6**). Comprehensive characterization by UV–vis, FT-IR, HRMS, NMR, EPR, electrochemistry, and single-crystal X-ray diffraction revealed that oxygenation exerts a clear influence on both macrocyclic structure and metal coordination. In particular, complex **6** features an unusual intramolecular V–O–C chelation motif arising from oxygenation at the inner carbon site, highlighting a distinctive mode of metal–ligand cooperation in N-confused porphyrinoid chemistry.

The two vanadium complexes also exhibit differentiated oxygen atom transfer reactivity toward cyclohexene and thioanisole in the presence of H_2_O_2_. Although cyclohexene oxidation is complicated by incomplete mass balance, complex **6** affords higher combined yields of the quantified products than does **5** under identical conditions. In thioanisole oxidation, both complexes promote sulfur oxidation beyond the catalyst-free background reaction, with **6** showing a high sulfone fraction. Overall, this study expands the chemistry of oxygenated N-confused porphyrins by linking controlled macrocycle oxygenation to unusual vanadium coordination environments and differentiated oxygen transfer reactivity.

## 7 Experimental Section

### 7.1 General Information

All reagents and solvents were purchased from commercial suppliers (Sigma-Aldrich) and used without further purification unless otherwise noted. ^1^H, ^13^C, and ^19^F NMR spectra were recorded on Bruker Avance III HD 400 MHz and Bruker DRX 500 MHz spectrometers. Chemical shifts (δ) are reported in ppm relative to internal tetramethylsilane (TMS) for ^1^H and ^13^C NMR spectra and to CFCl_3_ for ^19^F NMR spectra. High-resolution mass spectra (HRMS) were acquired on a JMS-700 double-focusing mass spectrometer (JEOL, Tokyo, Japan) for HRMS­(FAB) and a JMST100LP AccuTOF LC-plus 4G mass spectrometer for HRMS­(ESI). UV–vis spectra were recorded on an Agilent 8453 spectrophotometer. EPR spectra were collected at X-band frequency (9.5 GHz) with 100 kHz field modulation on a Bruker EMX spectrometer equipped with an ST4102 cavity under solid-state conditions at 77 K.

Single-crystal X-ray diffraction data were collected on a Bruker AXS APEX CCD diffractometer using graphite-monochromated Mo Kα radiation (λ = 0.71073 Å). The data were processed using SHELXL-97 with full-matrix least-squares refinement on F^2^. All nonhydrogen atoms were refined anisotropically, whereas hydrogen atoms were placed in idealized positions and refined using a riding model. Crystallographic data for compounds **3**, **4**, **5**, and **6** have been deposited with the Cambridge Crystallographic Data Centre (CCDC) under deposition numbers 2543033, 2542899, 2542897, and 2542890, respectively. These data are available from the CCDC.

### 7.2 Safety Statement

Hydrogen peroxide (30 wt %) is a strong oxidizing reagent and can cause severe skin burns and eye damage. Reactions involving H_2_O_2_ were carried out on the small scales described in Section 7 in a well-ventilated fume hood using appropriate personal protective equipment. H_2_O_2_ was kept away from combustible materials, reducing agents, and metal contaminants, and the reaction mixtures were not sealed. Waste containing residual oxidants was disposed of according to institutional procedures for oxidizing organic waste.

### 7.3 Synthesis of 5,10,15,20-Tetrakis­(pentafluorophenyl)-3-oxo-N-confused Porphyrin (3-oxo-*H*_2_PFNCP) (**3**)

The synthesis of N-confused pentafluorophenylporphyrin (*H*_2_PFNCP, **2**) and 3-oxo-*H*_2_PFNCP (**3**) was carried out according to a modified literature procedure.^42^ In a 2 L round-bottomed flask, CH_2_Cl_2_ (1.5 L), pyrrole (0.693 mL, 10 mmol), and pentafluorobenzaldehyde (1.23 mL, 10 mmol) were combined under N_2_. Trifluoromethanesulfonic acid (0.09 mL, 1 mmol) was added, and the mixture was stirred at room temperature for 24 h to give a dark solution. DDQ (2.50 g, 11.02 mmol) was then added, and after stirring for 1 min, the reaction mixture was quenched with triethylamine (0.5 mL, 4 mmol). The resulting mixture was stirred for an additional 30 min.

The solvent was removed under reduced pressure, and the residue was purified by silica gel chromatography (4 cm × 16 cm) using a gradient of *n*-hexane/CH_2_Cl_2_ (3:1 to 1:2). The compounds were eluted in the following order: *H*_2_TPFPP (**1**, 39%, 912 mg), *H*_2_PFNCP (**2**, 8.5%, 198 mg), and 3-oxo-*H*_2_PFNCP (**3**, 4.2%, 98 mg). The spectroscopic data for **1** and **2** were consistent with the reported values.^42^ Compound **3** was isolated here as a minor oxidation product.

#### 7.3.1 Compound **3** (3-oxo-*H*_2_PFNCP)

Yield 98 mg (0.010 mmol, 4.2%). ^1^H NMR (400 MHz, CDCl_3_) δ 8.85 (s, 1H, outer NH, broad), 8.81 (d, *J* = 5.0 Hz, 1H), 8.68 (d, *J* = 5.0 Hz, 1H), 8.65 (s, 2H), 8.61 (d, *J* = 4.7 Hz, 1H), 8.57 (d, *J* = 4.7 Hz, 1H), −2.80 (s, 2H, inner NH, broad), −5.47 (s, 1H, inner CH) (Figure S3). ^13^C NMR (101 MHz, CDCl_3_) δ 167.29, 155.94, 154.22, 147.65, 147.36, 147.19, 146.43, 146.24, 145.22, 145.17, 144.75, 144.54, 143.70, 143.53, 141.65, 139.59, 139.34, 138.93, 138.25, 137.35, 137.08, 136.89, 136.50, 136.37, 136.21, 135.95, 134.96, 134.24, 133.53, 127.69, 126.94, 126.72, 125.52, 124.75, 119.46, 115.22, 114.97, 113.16, 112.40, 111.20, 105.01, 102.74, 102.37, 94.37 (Figure S4). ^19^F NMR (376 MHz, CDCl_3_) δ −136.45 (s), −136.49 (s), −136.80 (d, *J* = 7.9 Hz), −136.86 (d, *J* = 7.0 Hz), −136.92 (d, *J* = 7.6 Hz), −139.49 (dd, *J* = 23.5, 7.5 Hz), −148.95 (t, *J* = 20.6 Hz), −151.17 (dt, *J* = 32.2, 20.9 Hz), −152.48 (t, *J* = 20.9 Hz), −159.22 (dt, *J* = 21.6, 10.9 Hz), −161.15 (dd, *J* = 28.9, 13.2 Hz), −162.06 (td, *J* = 22.9, 7.4 Hz) (Figure S7). UV–vis spectra (CH_3_CN) λ_max_ /nm (ε /M^–1^ cm^–1^): 319 (21743), 433 (170942), 528 (12966), 573 (7855), 630 (6210), 686 (4064) (Figure 3 and Table S2). ESI-HRMS (in acetonitrile): *m*/*z* = 989.0 for [M–H]^−^ (990.0 calcd for C_44_H_10_F_20_N_4_O) (Figure S13). FT-IR (Neat cm^–1^): 3373, 1706 correspond to NH, C=O stretching vibrations (Figures S1–S2 and Table S1).

### 7.4 Synthesis of Compounds **4**, **5**, and **6**

Compound **3** (100 mg, 0.10 mmol) and VO­(acac)_2_ (50 mg, 0.187 mmol) were dissolved in DMF (15 mL) and refluxed at 180 °C for 3 h. The solution gradually turned from light to dark green. After cooling, the solvent was removed under reduced pressure, and the residue was purified by silica gel chromatography (CH_2_Cl_2_/hexane gradient followed by MeOH/CH_2_Cl_2_). Three fractions were obtained: **4** (light green), 9% yield; **5** (bluish green), 35% yield; **6** (dark green), 30% yield. Single crystals of compound 6 suitable for X-ray diffraction were obtained by cocrystallization with tetrabutylammonium (TBA) salt from a suitable solvent system, which provides the countercation in the crystal structure.

#### 7.4.1 Compound **4** (3,21-dioxo-*H*_3_PFNCP)

Yield 9 mg (0.009 mmol, 9%).^1^H NMR (400 MHz, CDCl_3_) δ 8.05 (d, *J* = 4.7 Hz, 1H), 8.02 (d, *J* = 5.3 Hz, 1H), 7.86 (d, *J* = 4.9 Hz, 1H), 7.72 (d, *J* = 4.8 Hz, 1H), 7.69 (s, 1H), 7.66 (d, *J* = 4.5 Hz, 1H), 7.46 (d, *J* = 4.0 Hz, 1H), 6.92 (s, 1H, inner NH), 6.89 (s, 1H, inner NH), −0.35 (s, 1H, inner NH) (Figure S8). ^13^C NMR (101 MHz, CDCl_3_) δ 164.81, 163.39, 148.65, 147.52, 146.54, 145.10, 145.04, 144.01, 143.54, 141.24, 140.69, 139.61, 139.00, 138.60, 137.73, 137.43, 137.29, 137.12, 136.67, 136.52, 132.07, 130.30, 130.21, 129.13, 126.58, 124.93, 123.94, 119.94, 117.65, 117.53, 116.00, 113.88, 113.49, 113.44, 112.88, 112.58, 110.90, 110.68, 110.65, 110.26, 106.01, 101.64, 101.26, 91.82 (Figure S9). ^19^F NMR (376 MHz, CDCl_3_) δ −136.84 (dd, *J* = 21.1, 6.0 Hz), −137.15 (dd, *J* = 22.7, 7.1 Hz), −137.39 (dd, *J* = 21.8, 6.1 Hz), −139.80 (dd, *J* = 23.0, 7.1 Hz), −149.87 (t, *J* = 20.9 Hz), −150.54 (t, *J* = 20.8 Hz), −150.91 (t, *J* = 20.9 Hz), −152.84 (t, *J* = 20.9 Hz), −159.25 (dd, *J* = 20.8, 15.0 Hz), −160.46 (td, *J* = 21.8, 6.4 Hz), −162.00 (t, *J* = 18.7 Hz) (Figure S12). UV–vis spectra (CH_3_CN) λ_max_ /nm (ε /M^–1^ cm^–1^): 353 (40691), 450 (57727), 480 (54472), 585 (4001), 650 (4739), 790 (7992) (Figure 3 and Table S2). ESI-HRMS (in acetonitrile): *m*/*z* = 1006.0 for [M]^**+**^ (1006.05 calcd for C_44_H_10_F_20_N_4_O_2_) (Figure S14). FT-IR (Neat cm^–1^): 3290, 1703 correspond to NH, C=O stretching vibrations (Figures S1–S2 and Table S1).

#### 7.4.2 Compound **5** V­(=O)­(OH)­(3-oxo-PFNCP)

Yield 38 mg (0.035 mmol, 35%). UV–vis spectra (CH_3_CN) λ_max_ /nm (ε /M^–1^ cm^–1^): 297 (25837), 357 (25577), 436 (145610), 542 (7556), 584 (6352), 617 (10305), 672 (47991) (Figure 3 and Table S2). ESI-HRMS (in acetonitrile): *m*/*z* = 1069.9626 for [M–H]^−^ (1070.97 calcd for C_44_H_8_F_20_N_4_O_3_V) (Figure S15). FT-IR (Neat cm^–1^): 445, 951, 3290, 1733 correspond to V–OH, V=O, NH, C=O stretching vibrations (Figures S1–S2 and Table S1).

#### 7.4.3 Compound **6** [*n*-Bu_4_N]^+^[V­(OH)­(3,21-dioxo-PFNCP)]^−^

Yield 32 mg (0.030 mmol, 30%). UV–vis spectra (CH_3_CN) λ_max_ /nm (ε /M^–1^ cm^–1^): 315 (24389), 352 (21981), 414 (27568), 471 (62632), 488 (44311), 674 (9714), 716 (8731) (Figure 3 and Table S2). ESI-HRMS (in acetonitrile): *m*/*z* = 1069.96 for [M–H]^−^ (1070.97 calcd for C_44_H_7_F_20_N_4_O_3_V) (Figure S16). FT-IR (Neat cm^–1^): 487, 755, 1695 correspond to V–O–C, V–OH, C=O stretching vibrations (Figures S1–S2 and Table S1).

### 7.5 Catalytic Reactions

#### 7.5.1 Cyclohexene Oxidation

Catalytic tests were conducted in CH_3_CN (5 mL) containing catalyst (0.002 M), cyclohexene (0.125 M), and H_2_O_2_ (0.2 M). The reaction mixture was stirred at 45 °C under air. Aliquots (1 mL) were withdrawn at the indicated time intervals and analyzed by GC-FID using a PerkinElmer AutoSystem XL gas chromatograph equipped with a Restek Stabilwax-DA column (30 m × 0.25 mm i.d., 0.5 μm film thickness). Quantification was performed using calibration curves obtained from authentic standards (Figure S29). Product distributions were determined from the relative peak areas of cyclohexene and the corresponding oxidation products.

#### 7.5.2 Thioanisole Oxidation

Analogous reactions were carried out in CH_3_CN (10 mL) containing a catalyst (0.0006 M), thioanisole (0.125 M), and H_2_O_2_ (0.65 M) at room temperature for 20 h. The products were analyzed by GC-FID under identical conditions. Calibration with authentic thioanisole, sulfoxide, and sulfone standards enabled quantitative evaluation of conversion and selectivity (Figure S30).

### 7.6 Computational Methods

All calculations were performed using the Gaussian 16 software package.^43^ Geometry optimizations of compounds **3–6** were carried out at the **B3LYP/6-311G­(d,p)** level of theory, with the **LANL2DZ** pseudopotential applied to the vanadium center. For compound **6**, DFT calculations were conducted on the isolated anionic complex without the counterion.

## Supplementary Material



## Notes

^1^H, ^13^C, and ^19^F NMR spectra; COSY and HSQC spectra; ESI-MS and HR-ESI-MS spectra; UV–vis and emission spectra; cyclic voltammetry data; DFT-optimized geometries and frontier molecular orbitals; calibration curves; catalytic studies; and optimized Cartesian coordinates for compounds **3**–**6** (PDF)
