# Supplementary material for: Oxygenated N‑Confused Porphyrins: Structural Elucidation and Distinct Vanadium(IV) Coordination Motifs
Source: Inorg Chem. 2026 Jun 18;65(26):14880–91. doi: 10.1021/acs.inorgchem.6c01709 (PMC13343513; doi:10.1021/acs.inorgchem.6c01709)
Supplement: Supplementary file 1 [file ic6c01709_si_001.pdf]

Supporting Information

Oxygenated N-Confused Porphyrins: Structural Elucidation and Distinct Vanadium(IV) Coordination Motifs

Bhakyaraj Kasi[a,b,c], Tanmoy Pain[a], Kai-Chun Hsu[a,d,e], Jian-Hong Liao[a], Chen-Hsiung Hung[a,f,g]*

[a] Institute of Chemistry, Academia Sinica, Nankang Taipei 115201, Taiwan

[b] Department of Chemistry, National Tsing Hua University, Hsinchu 300044, Taiwan

[c] Molecular Science and Technology Program, Taiwan International Graduate Program, Academia Sinica, Taipei 115201, Taiwan

[d] Nano Science and Technology Program, Taiwan International Graduate Program, Academia Sinica, Taipei 115201, Taiwan

[e] Department of Chemistry, National Taiwan University, Taipei 106319, Taiwan

[f] Department of Chemistry, National Taiwan Normal University, Taipei 116059, Taiwan

[g] Department of Medicinal and Applied Chemistry, Kaohsiung Medical University, Kaohsiung 807378, Taiwan

E-mail: chhung@gate.sinica.edu.tw

CONTENT	Page NO.
**Figure S1** FT-IR Spectrum of (a) (**3**); (b) (**4**)**;** (c) (**5**) and (d) (**6)** as a KBr pellet.	**S4**
**Figure S2** FT-IR Spectrum of (a) (**3**); (b) (**4**)**;** (c) (**5**) and (d) (**6)** from the DFT.	**S5**
**Figure S3** ^{1}H NMR (400 MHz) spectrum of **3** in $CDCl_3$.	**S6**
**Figure S4** ^{13}C NMR (101 MHz) spectrum of **3** in $CDCl_3$.	**S7**
**Figure S5** ^{1}H-^{1}H COSY (400 MHz) spectrum of **3** in $CDCl_3$.	**S8**
**Figure S6** ^{1}H-^{13}C HSQC (400 MHz) spectrum of **3** in $CDCl_3$.	**S9**
**Figure S7** ^{19}F NMR (376 MHz) spectrum of **3** in $CDCl_3$.	**S10**
**Figure S8** ^{1}H NMR (400 MHz) spectrum of **4** in $CDCl_3$.	**S11**
**Figure S9** ^{13}C NMR (101 MHz) spectrum of **4** in $CDCl_3$.	**S12**
**Figure S10** ^{1}H-^{1}H COSY (400 MHz) spectrum of **4** in $CDCl_3$.	**S13**
**Figure S11** ^{1}H-^{13}C HSQC (400 MHz) spectrum of **4** in $CDCl_3$.	**S14**
**Figure S12** ^{19}F NMR (376 MHz) spectrum of **4** in $CDCl_3$.	**S15**
**Figure S13** ESI- MS spectrum of **3** in CH_3CN.	**S16**
**Figure S14** ESI- MS spectrum of **4** in CH_3CN.	**S17**
**Figure S15** ESI- MS spectrum of **5** in CH_3CN.	**S18**
**Figure S16** ESI- MS spectrum of **6** in CH_3CN.	**S19**
**Figure S17** Emission spectrum of **3** and **4** in CH_3CN.	**S20**
**Figure S18** Cyclic voltammograms of compounds (a) **3** and (b) **4** respectively, recorded in CH_2Cl_2 with potentials referenced to the Fc^0/Fc^+ couple.	**S21**
**Figure S19** DFT- optimized geometry of 3-oxo-*H₂*PFNCP,**3** using the 6-311G (d, p) basis set.	**S22**
**Figure S20** Selected frontier MOs, along with their orbital energies of the 3-oxo-H_2PFNCP,**3.**	**S23**
**Figure S21** DFT- optimized geometry of 3,21-dioxo-H_3PFNCP,**4** using the 6-311G (d, p) basis set.	**S24**
**Figure S22** Selected frontier MOs, along with their orbital energies of the 3,21-dioxo-H_3PFNCP,**4**	**S25**
**Figure S23** DFT- optimized geometry of V(=O)(OH)(3-oxo-PFNCP),**5** using the 6-311G (d, p) basis set.	**S26**
**Figure S24** Selected frontier MOs, along with their orbital energies of the V(=O)(OH)(3-oxo-PFNCP),**5**	**S27**

**Figure S25** DFT- optimized geometry of $[n\text{-}BnN_4]^+$**[**V(OH)(3,21-dioxo-PFNCP)$]^-$,**6** using the 6-311G (d, p) basis set.	**S28**
**Figure S26** Selected frontier MOs, along with their orbital energies of the $[n\text{-}BnN_4]^+$**[**V(OH)(3,21-dioxo-PFNCP)$]^-$,**6**	**S29**
**Figure S27** Electronic Absorption Spectra of the reaction mixture of cyclohexene oxidation.	**S30**
**Figure S28a** HR-ESI- MS spectrum the reaction mixture of cyclohexene oxidation **(0-8h)**	**S31**
**Figure S28b** LR-ESI- MS spectrum the reaction mixture of cyclohexene oxidation **(4-8h)**	**S32**
**Figure S29** Calibration curves were generated for cyclohexene (a), cyclohexene oxide (b), 2-cyclohexen-1-ol (c), and cyclohexen-1-one (d) using hexane as the solvent. Respectively, calibration curves for cyclohexane-1,2-diol (e) were established using acetonitrile.	**S33**
**Figure S30** Calibration curves were generated for $PhSCH_3$(a), $PhSOCH_3$ (b), and $PhSO_2CH_3$ (c) using methanol as the solvent.	**S34**
**Table S1** FT-IR Stretching Frequency of all the compounds.	**S35**
**Table S2** UV-vis. data of all the compounds.	**S36**
**Table S3** Experimental and Theoretical bond distances.	**S37**
**Table S4** Catalysis study of cyclohexene with compound **5**.	**S38**
**Table S5** Catalysis study of cyclohexene with compound **6**.	**S39**
**Table S6** Catalysis study of Thioanisole with H_2O_2.	**S40**
**Table S7** Catalysis study of Thioanisole with compound **5**.	**S41**
**Table S8** Catalysis study of Thioanisole with compound **6**.	**S42**
Optimized Cartesian Co-ordinates of 3-oxo-H_2PFNCP, **3**	**S43-S45**
Optimized Cartesian Co-ordinates of 3,21-dioxo-H_3PFNCP, **4**	**S46-S48**
Optimized Cartesian Co-ordinates of V(=O)(OH)(3-oxo-PFNCP), **5**	**S49-S51**
Optimized Cartesian Co-ordinates of $[n\text{-}BnN_4]^+$**[**V(OH)(3,21-dioxo-PFNCP)$]^-$, **6**	**S52-S54**

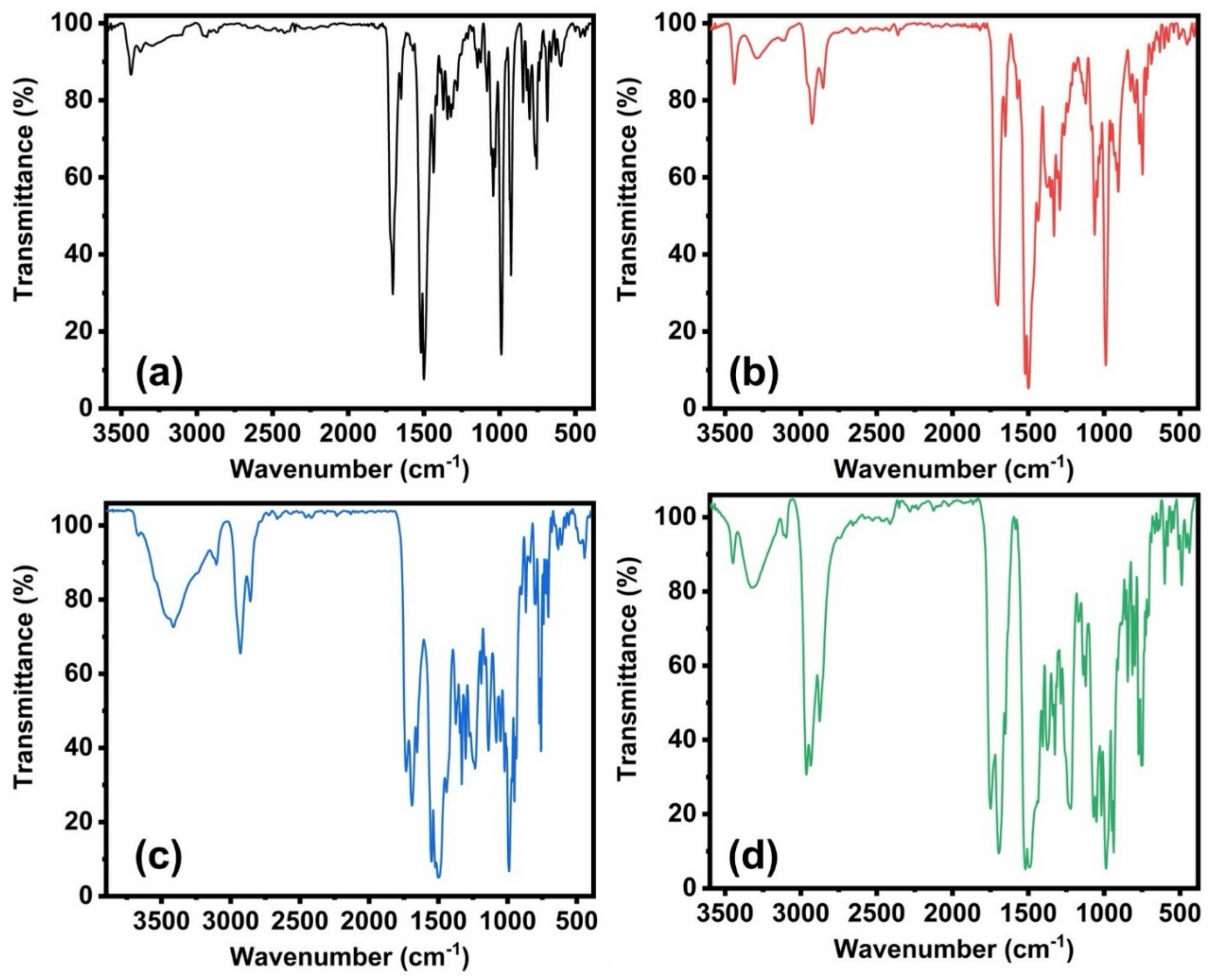

Figure S1 FT-IR Spectrum of (a) (**3**); (b) (**4**); (c) (**5**) and (d) (**6**) as a KBr pellet.

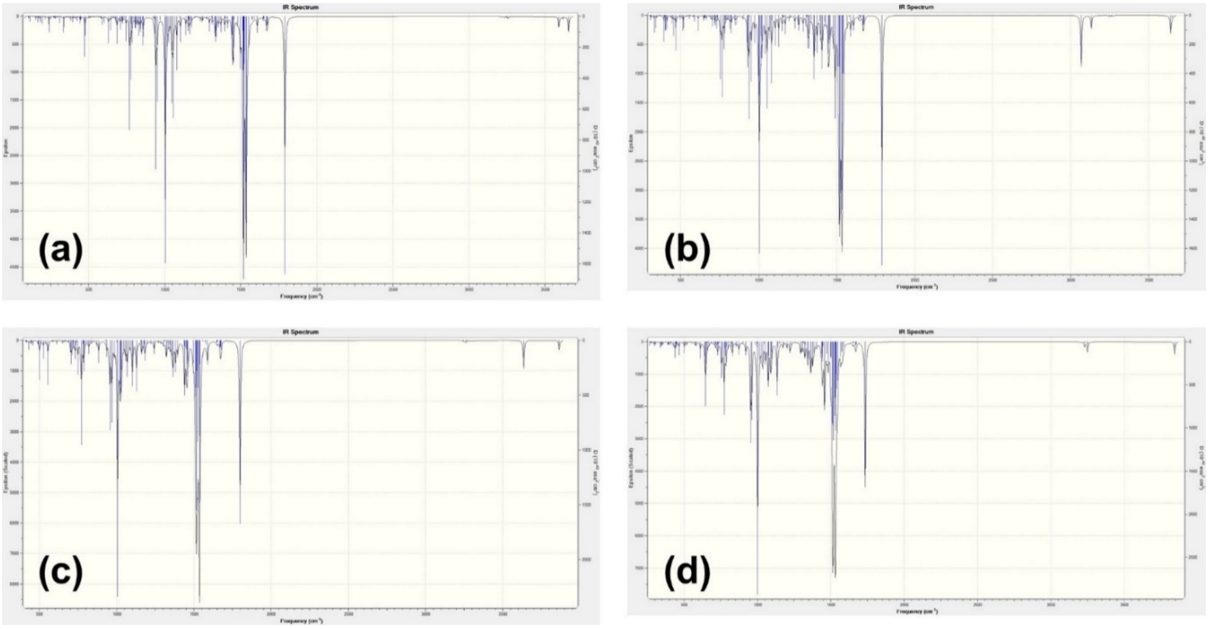

Figure S2 FT-IR Spectrum of (a) (**3**); (b) (**4**)**;** (c) (**5**) and (d) **(6)** from the DFT.

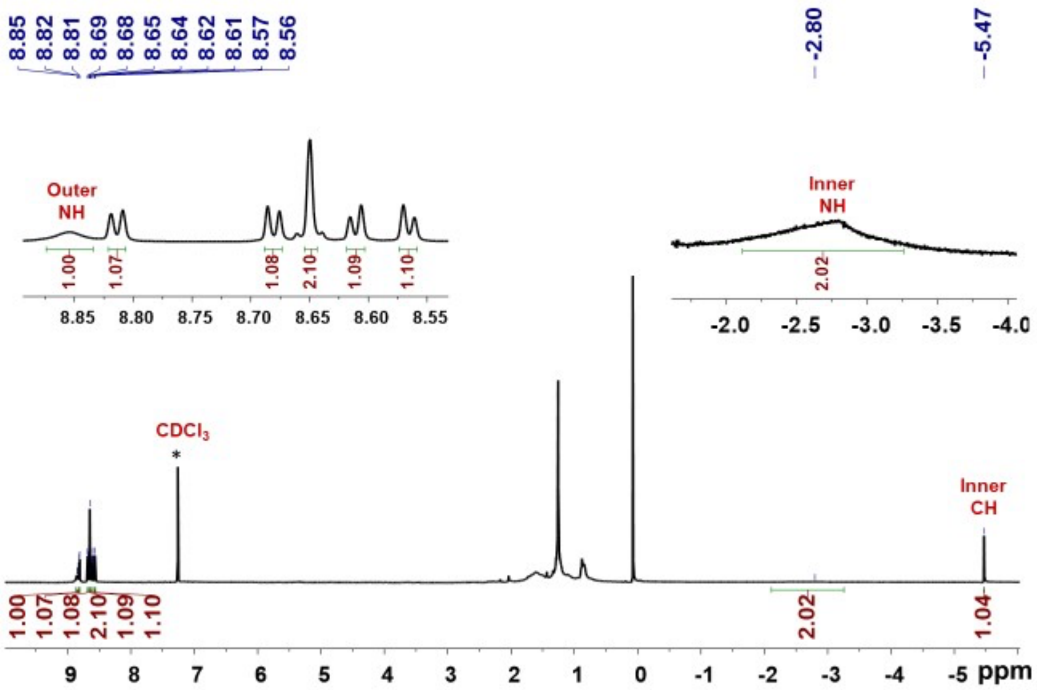

Figure S3 ^{1}H NMR (400 MHz) spectrum of **3** in $CDCl_3$.

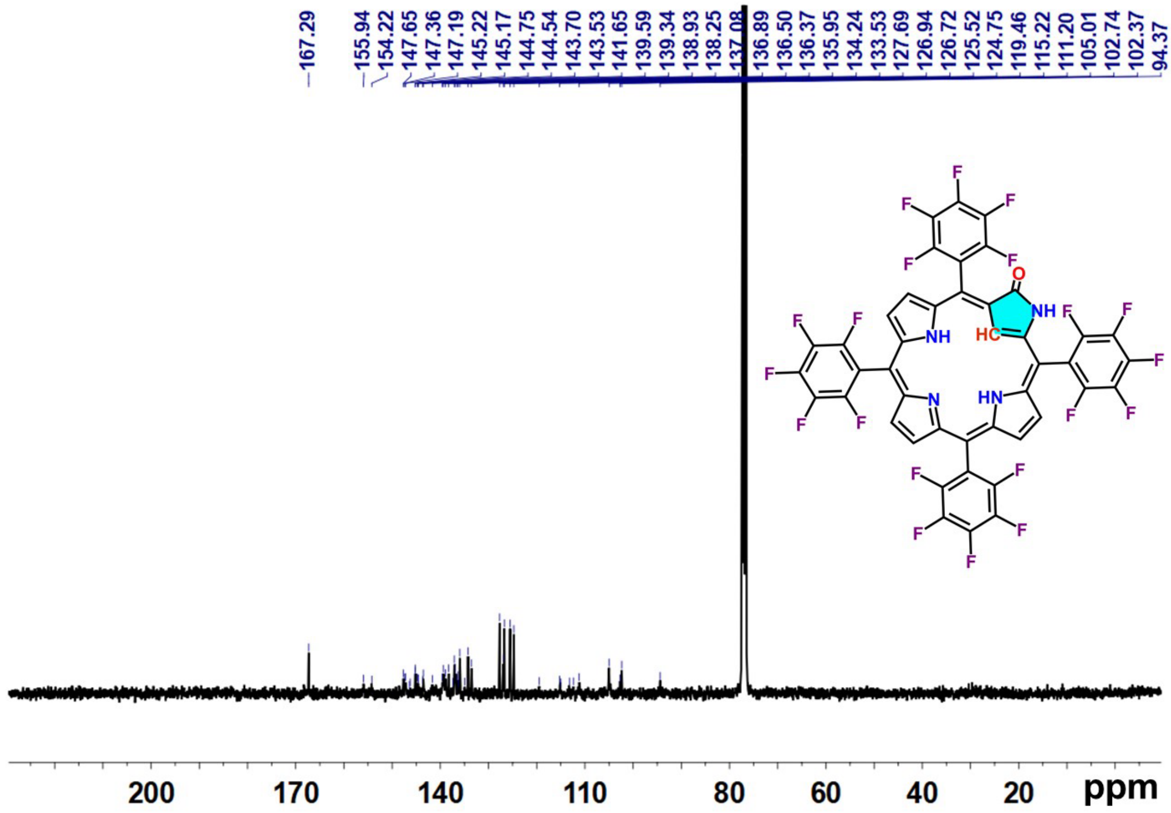

Figure S4 ^{13}C NMR (101 MHz) spectrum of **3** in $CDCl_3$.

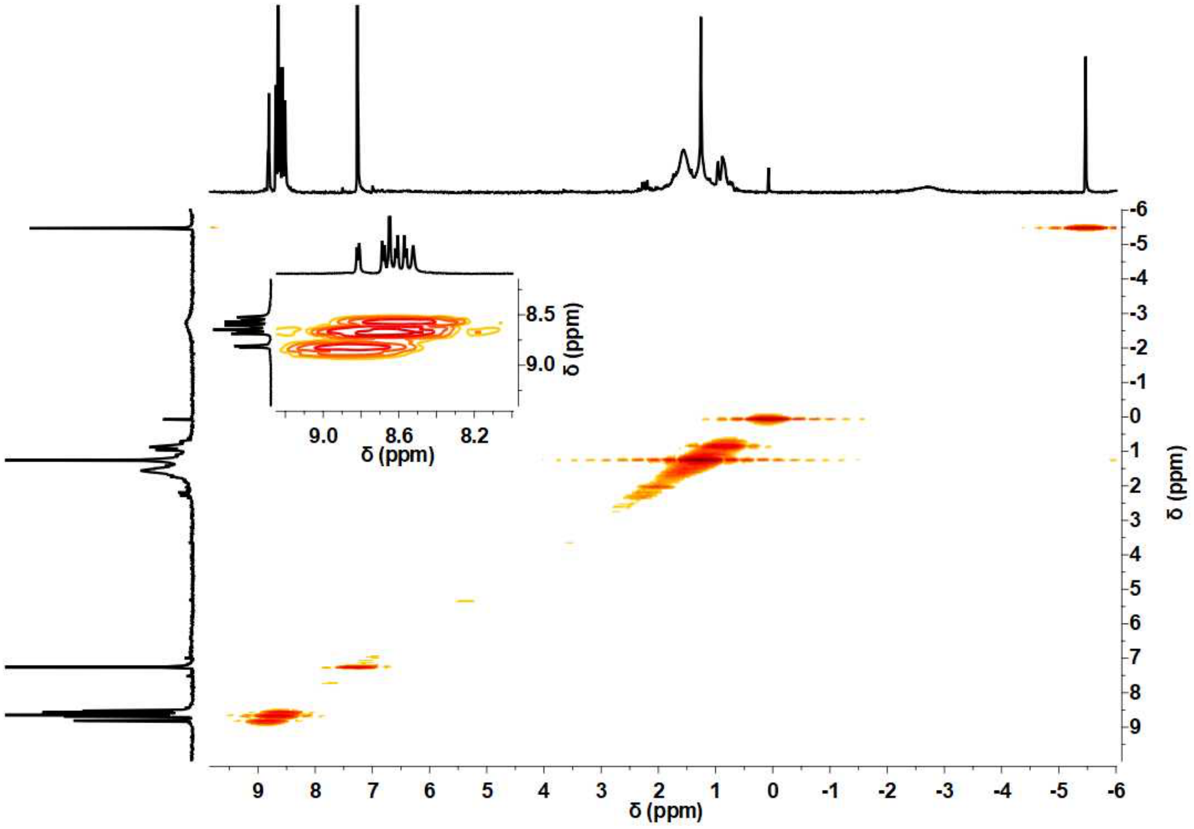

Figure S5 ^1H-^1H COSY (400 MHz) spectrum of **3** in $CDCl_3$.

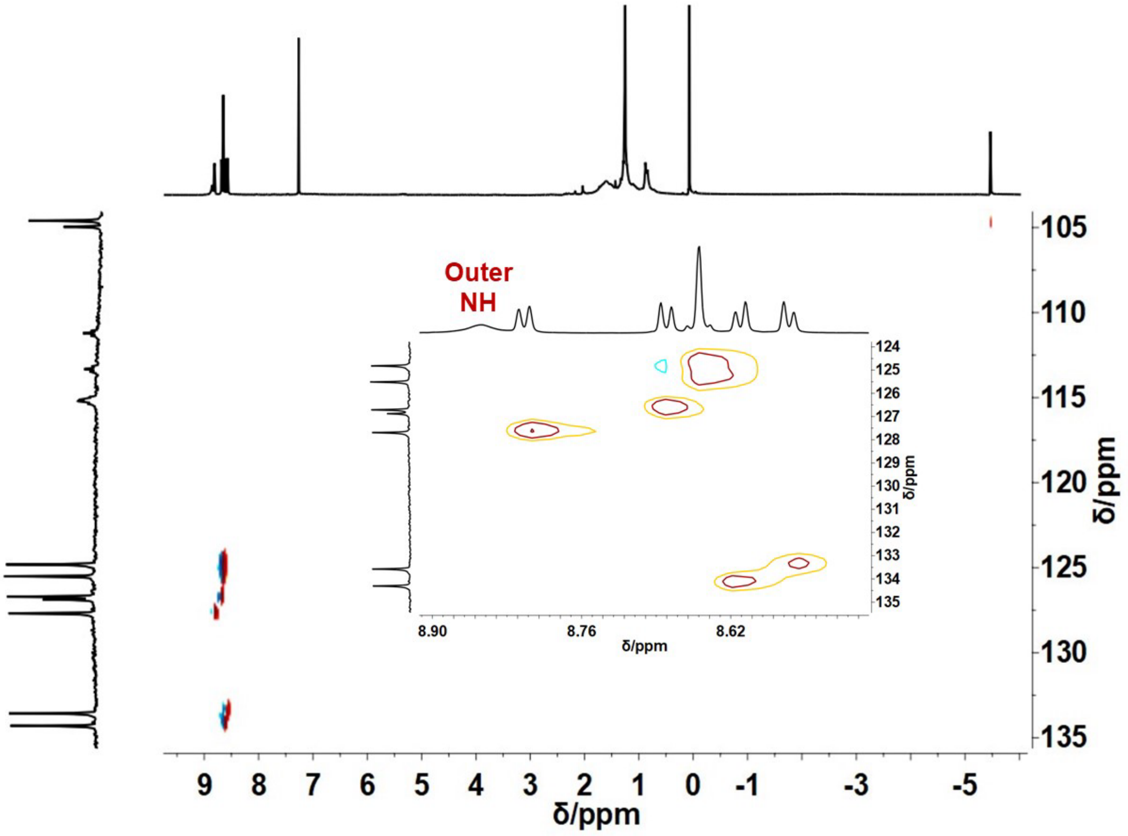

Figure S6 ^{1}H-^{13}C HSQC (400 MHz) spectrum of **3** in $CDCl_3$.

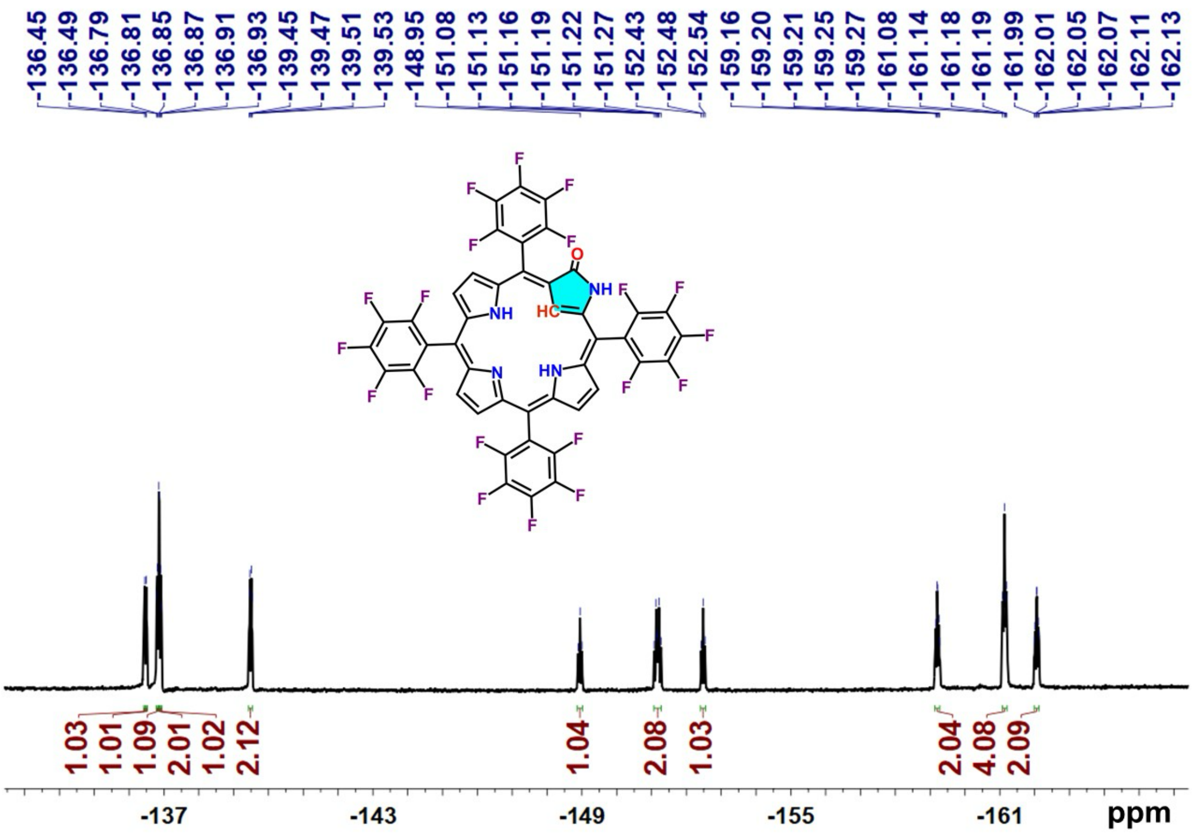

Figure S7 ^{19}F NMR (376 MHz) spectrum of **3** in $CDCl_3$.

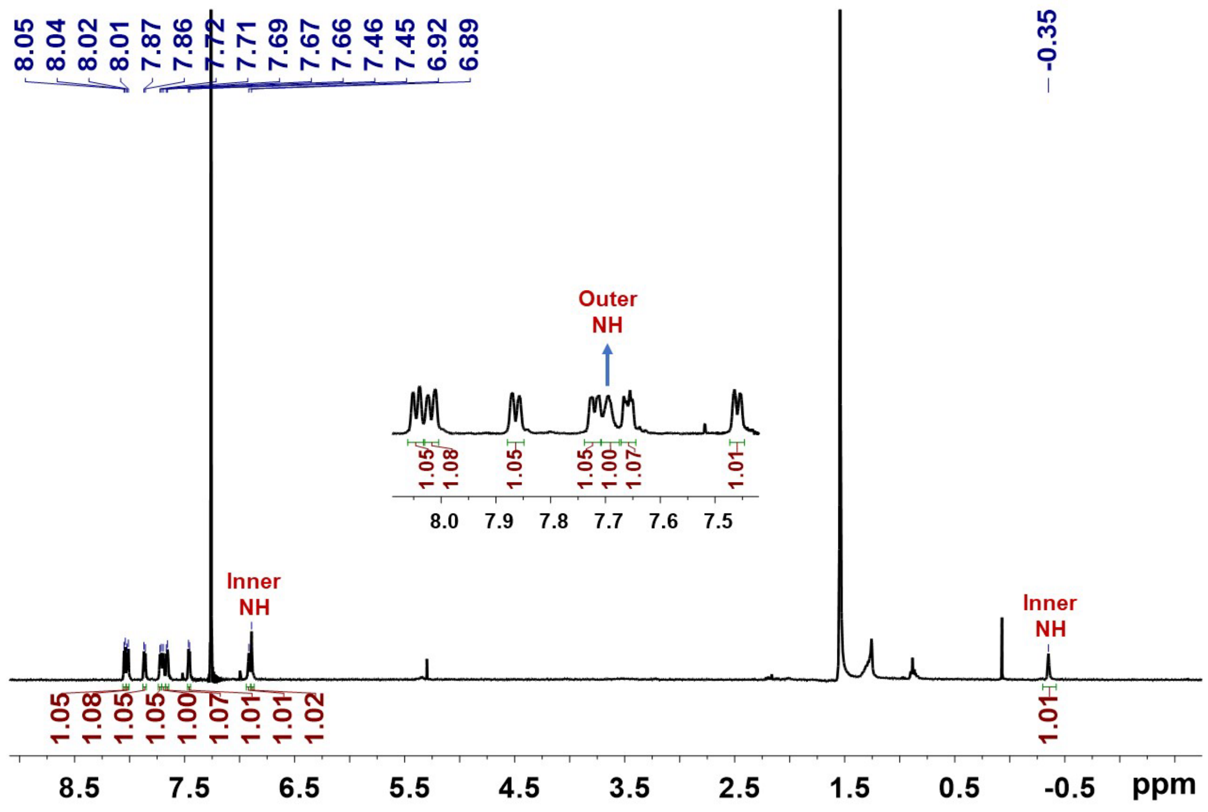

Figure S8 ^{1}H NMR (400 MHz) spectrum of **4** in $CDCl_3$.

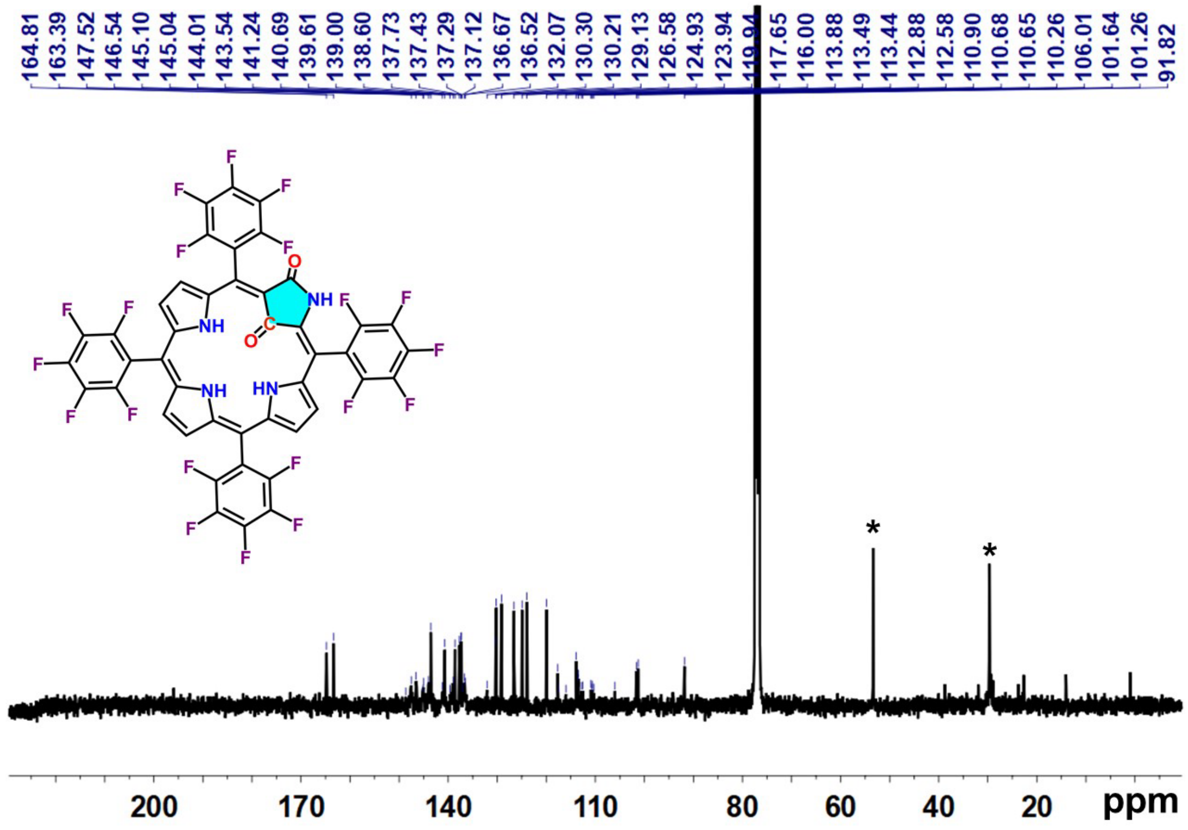

Figure S9 ^{13}C NMR (101 MHz) spectrum of **4** in $CDCl_3$.

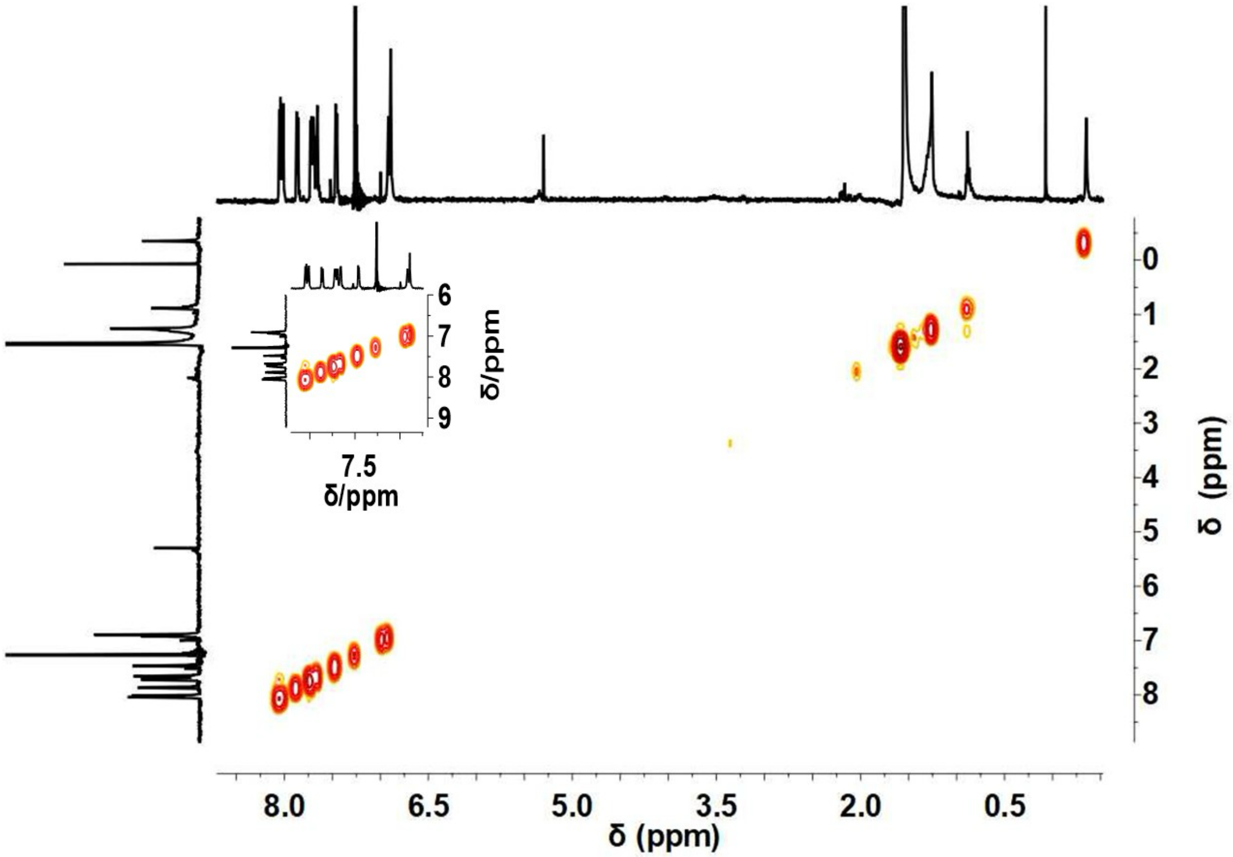

Figure S10 ^{1}H-^{1}H COSY (400 MHz) spectrum of **4** in $CDCl_3$.

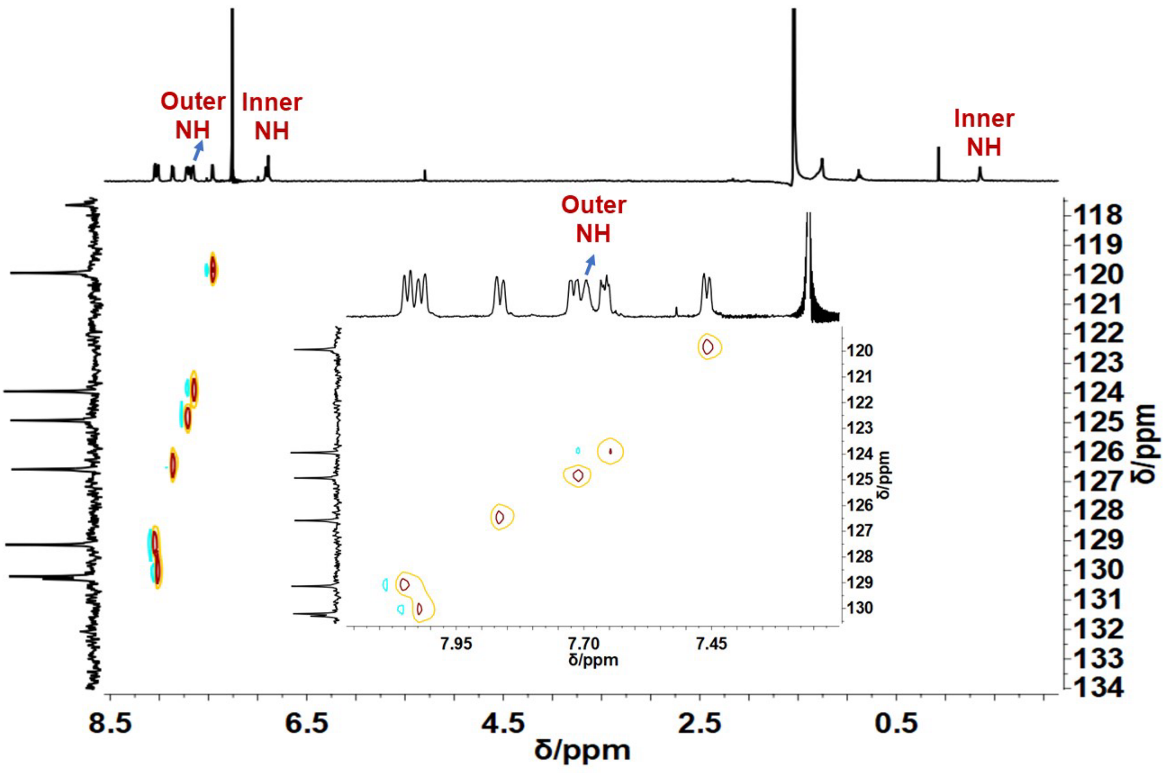

Figure S11 ^{1}H-^{13}C HSQC (400 MHz) spectrum of **4** in $CDCl_3$.

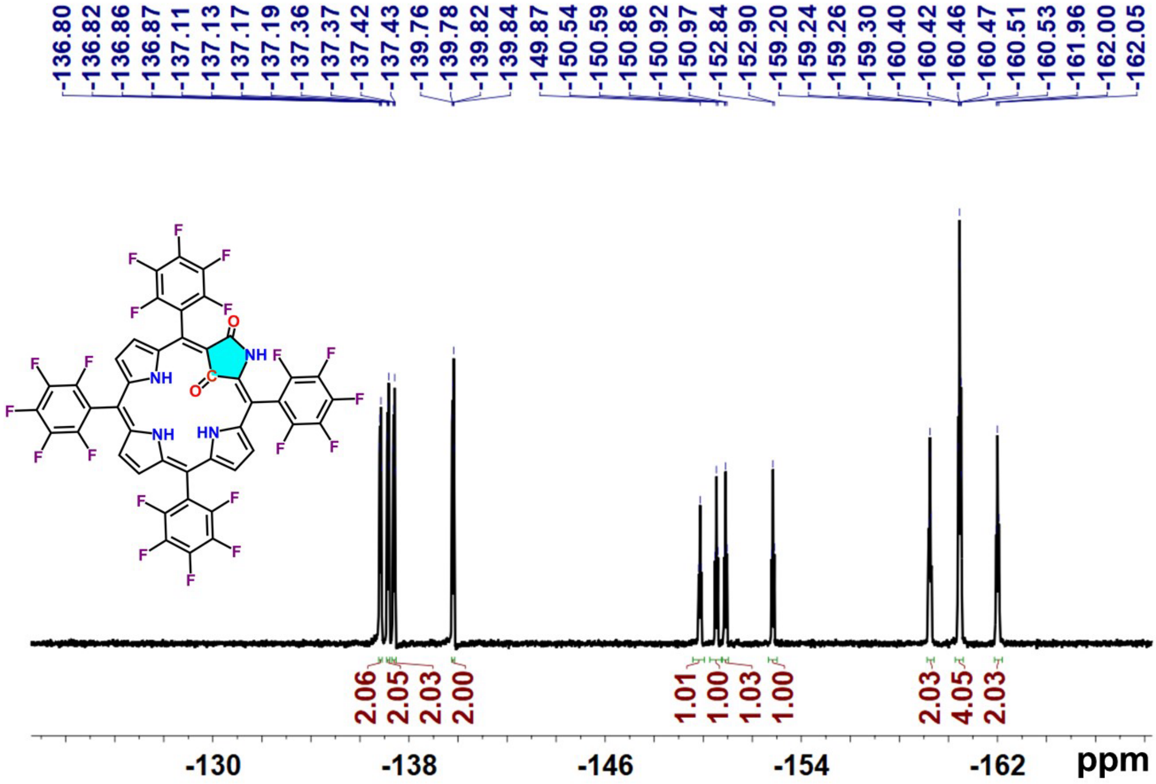

Figure S12 ^{19}F NMR (376 MHz) spectrum of **4** in $CDCl_3$.

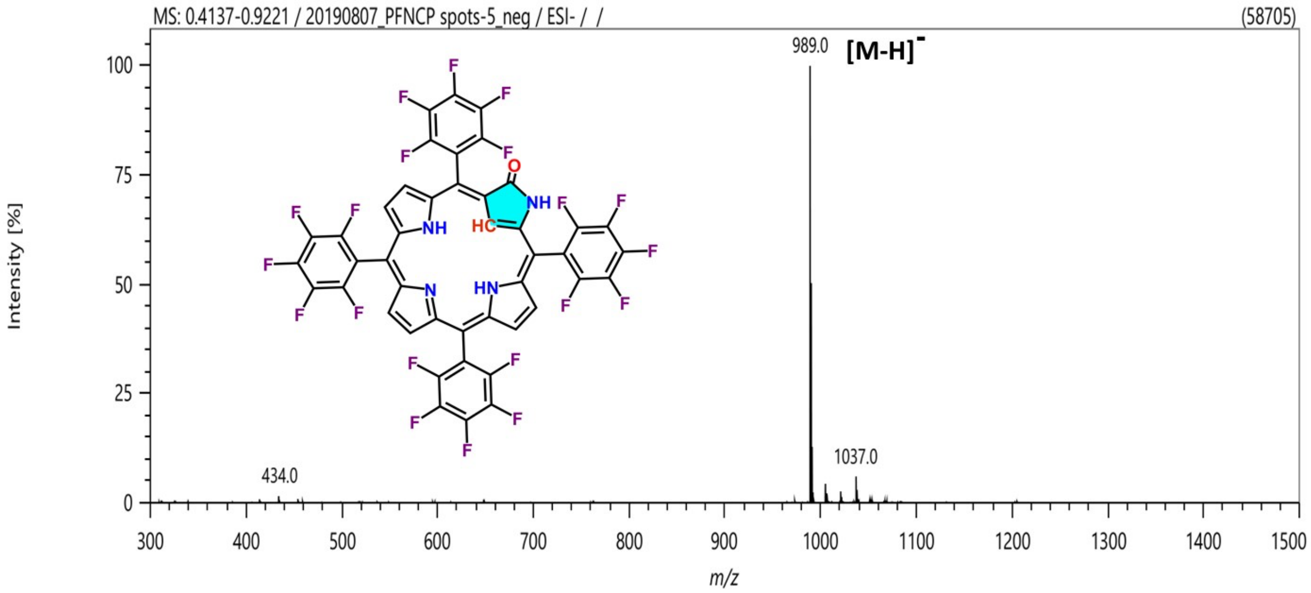

Figure S13 ESI- MS spectrum of **3** in CH_3CN.

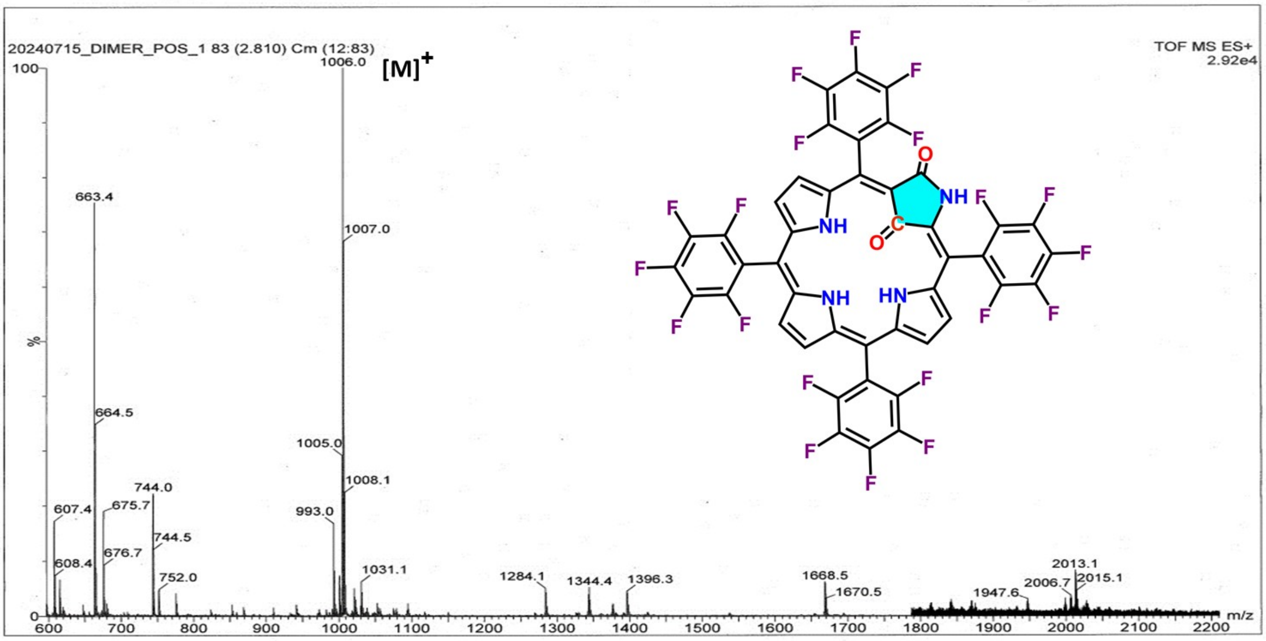

Figure S14 ESI- MS spectrum of **4** in CH_3CN.

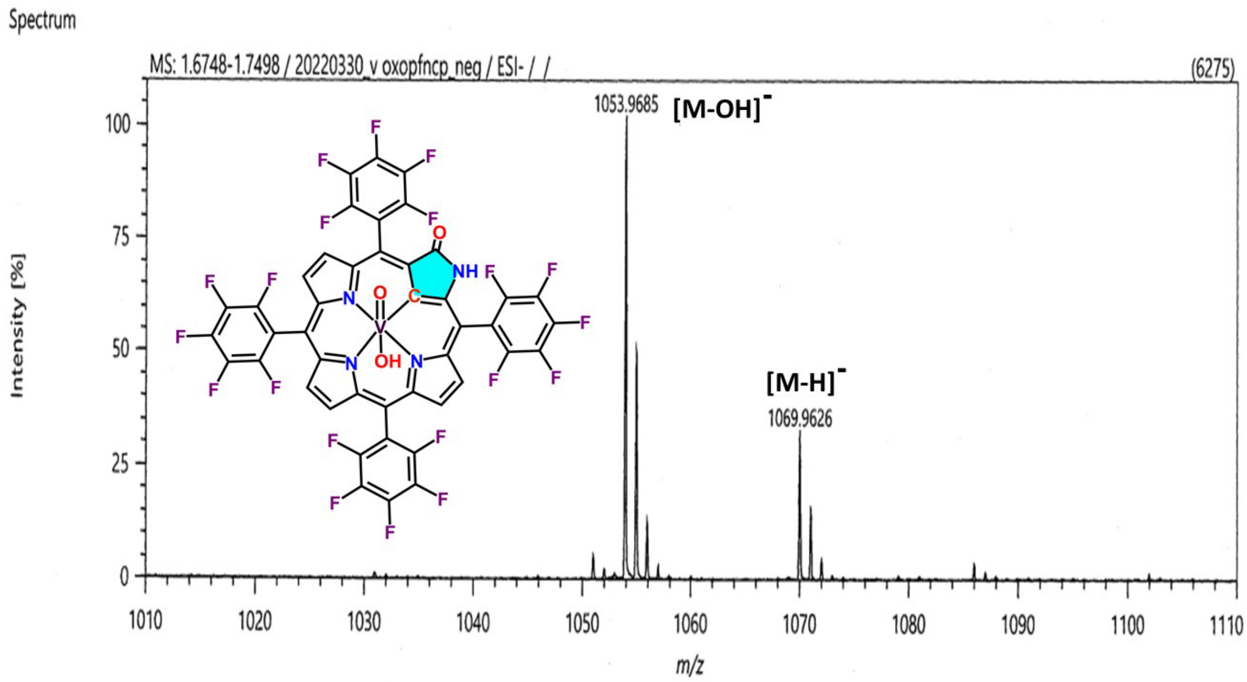

Figure S15 ESI- MS spectrum of **5** in CH_3CN.

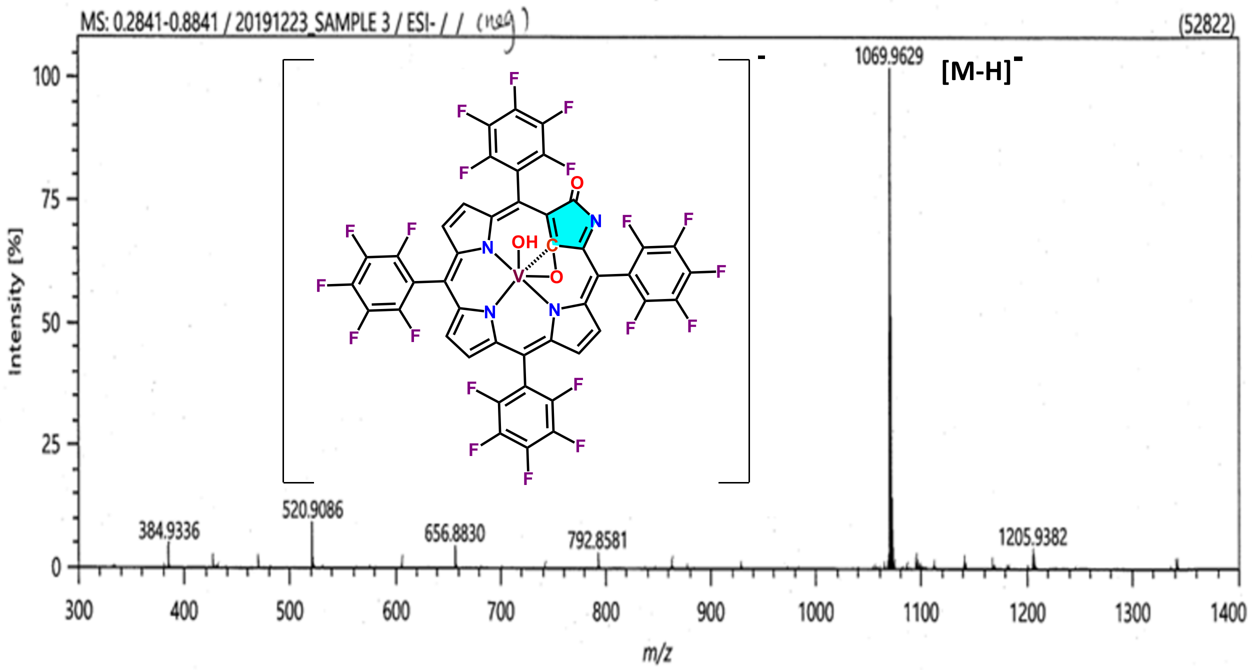

Figure S16 ESI-MS spectrum of **6** in CH_3CN.

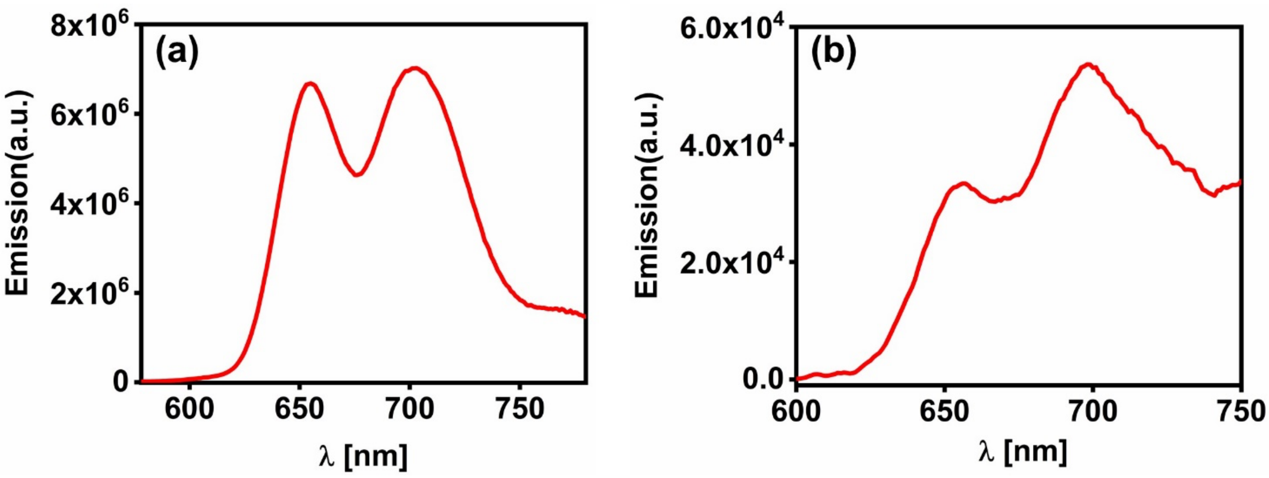

Figure S17 Emission spectrum of (a) **3** and (b) **4** in CH_3CN.

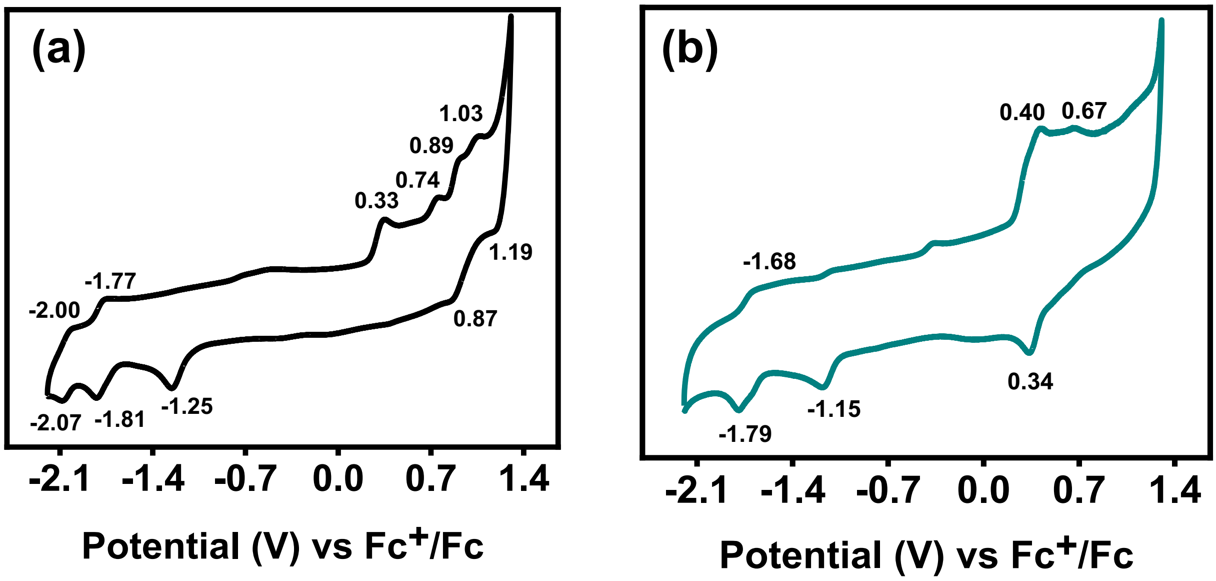

Figure S18 Cyclic voltammograms of compounds (a) **3** and (b) **4** respectively, recorded in CH_2Cl_2 with potentials referenced to the Fc^0/Fc^+ couple.

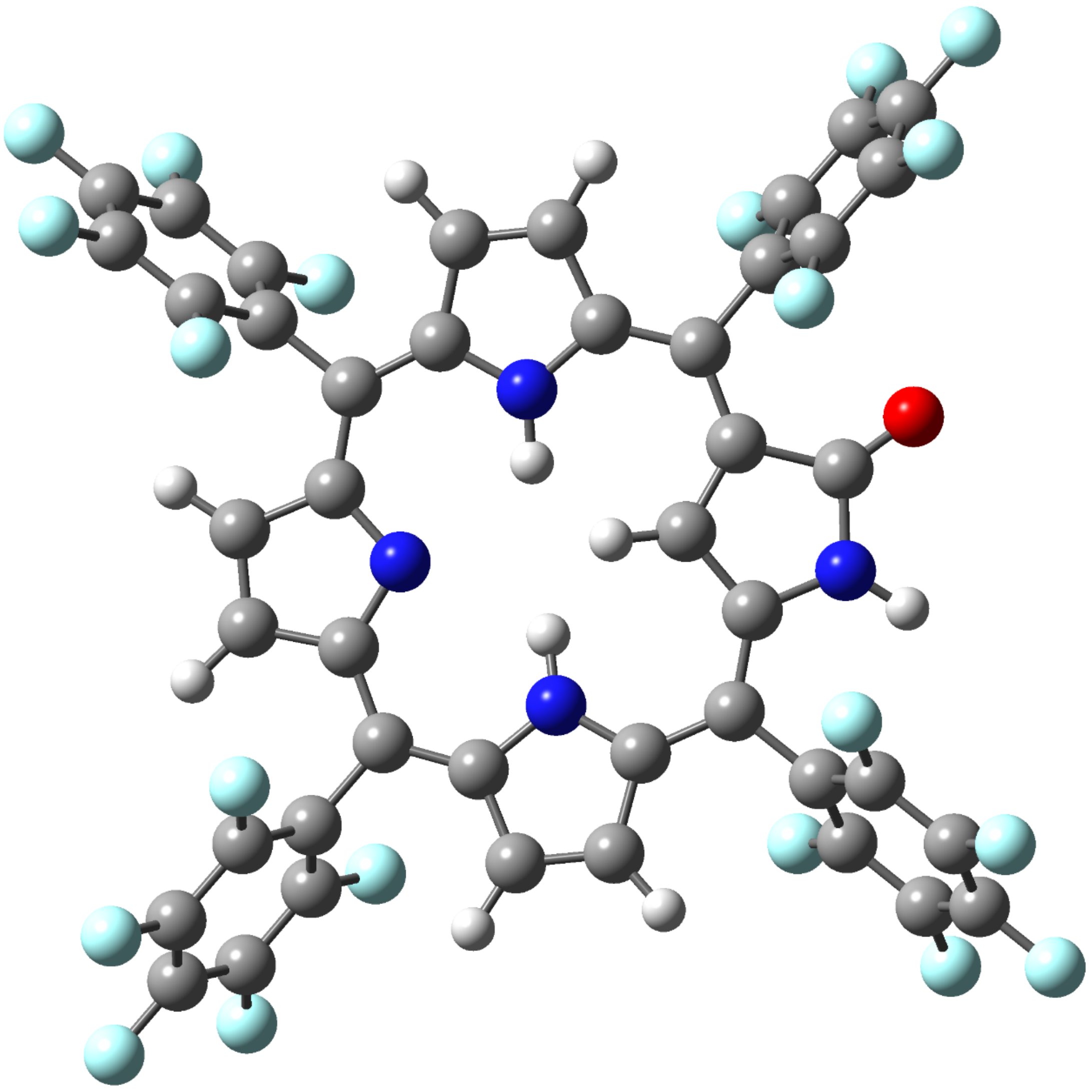

Figure S19 DFT- optimized geometry of 3-oxo-*H*$_2$PFNCP,**3** using the 6-311G (d, p) basis set.

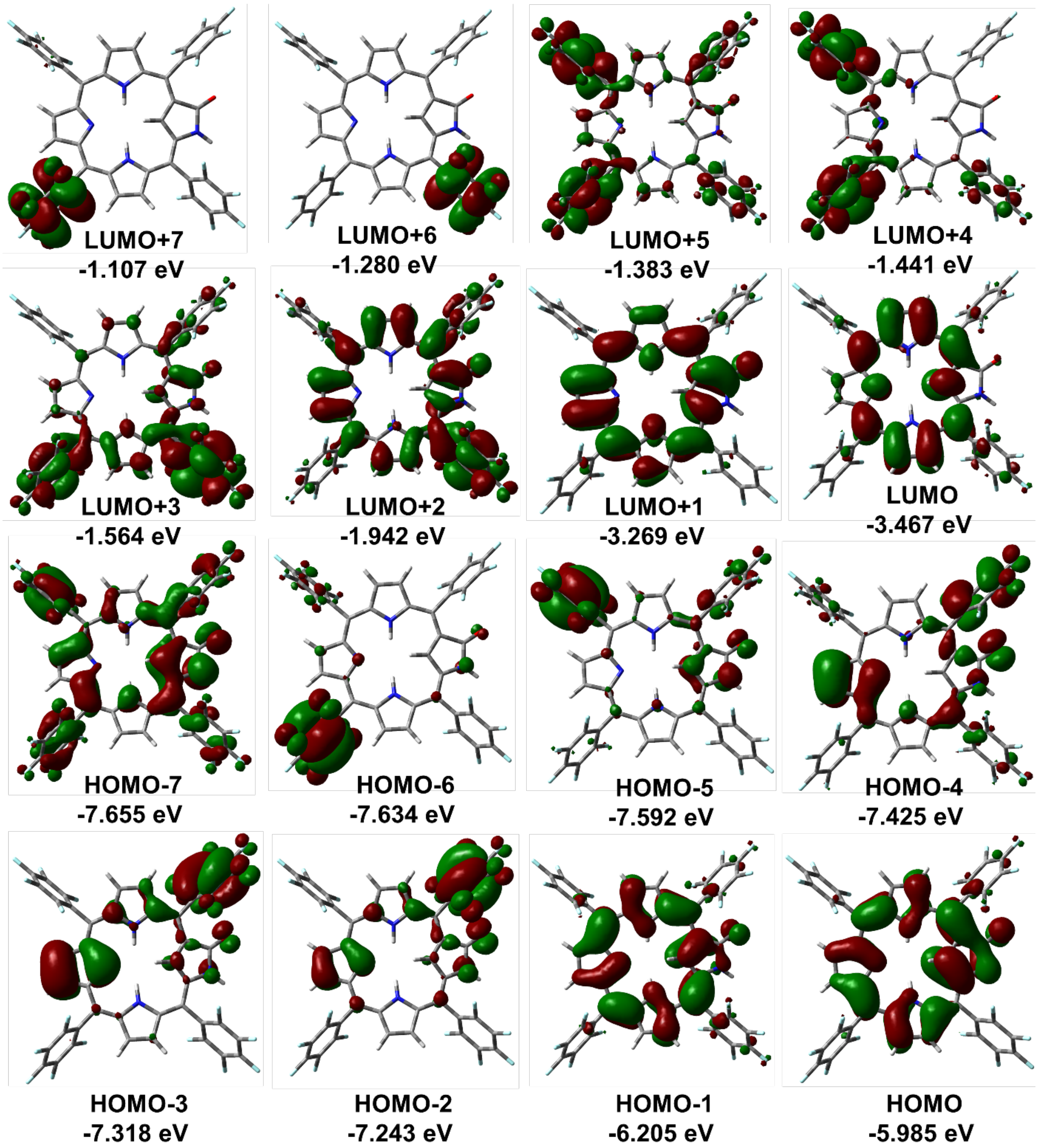

Figure S20 Selected frontier MOs, along with their orbital energies of the 3-oxo-*H_2*PFNCP,**3.**

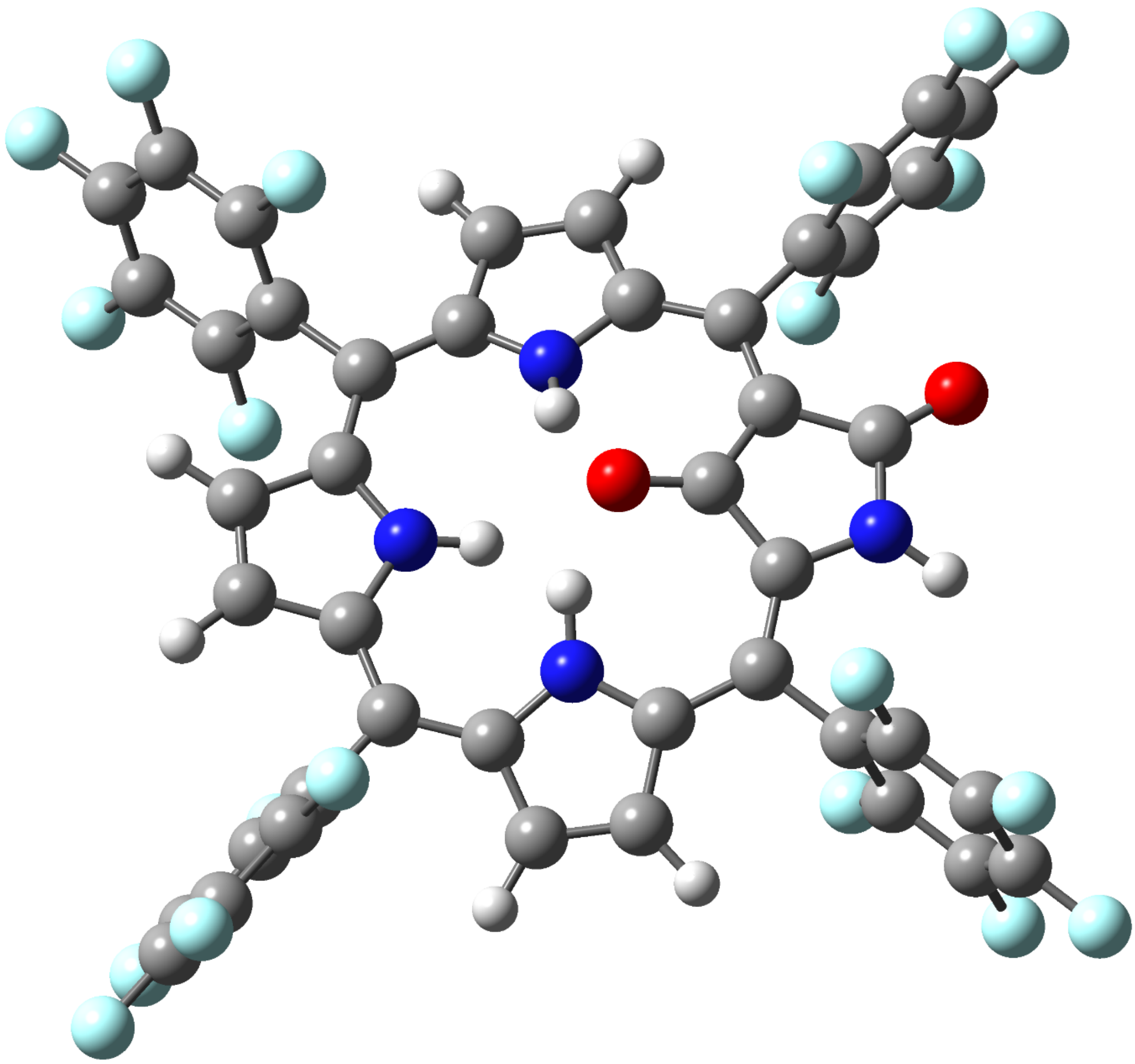

Figure S21 DFT- optimized geometry of 3,21-dioxo-H_3PFNCP,**4** using the 6-311G (d, p) basis set.

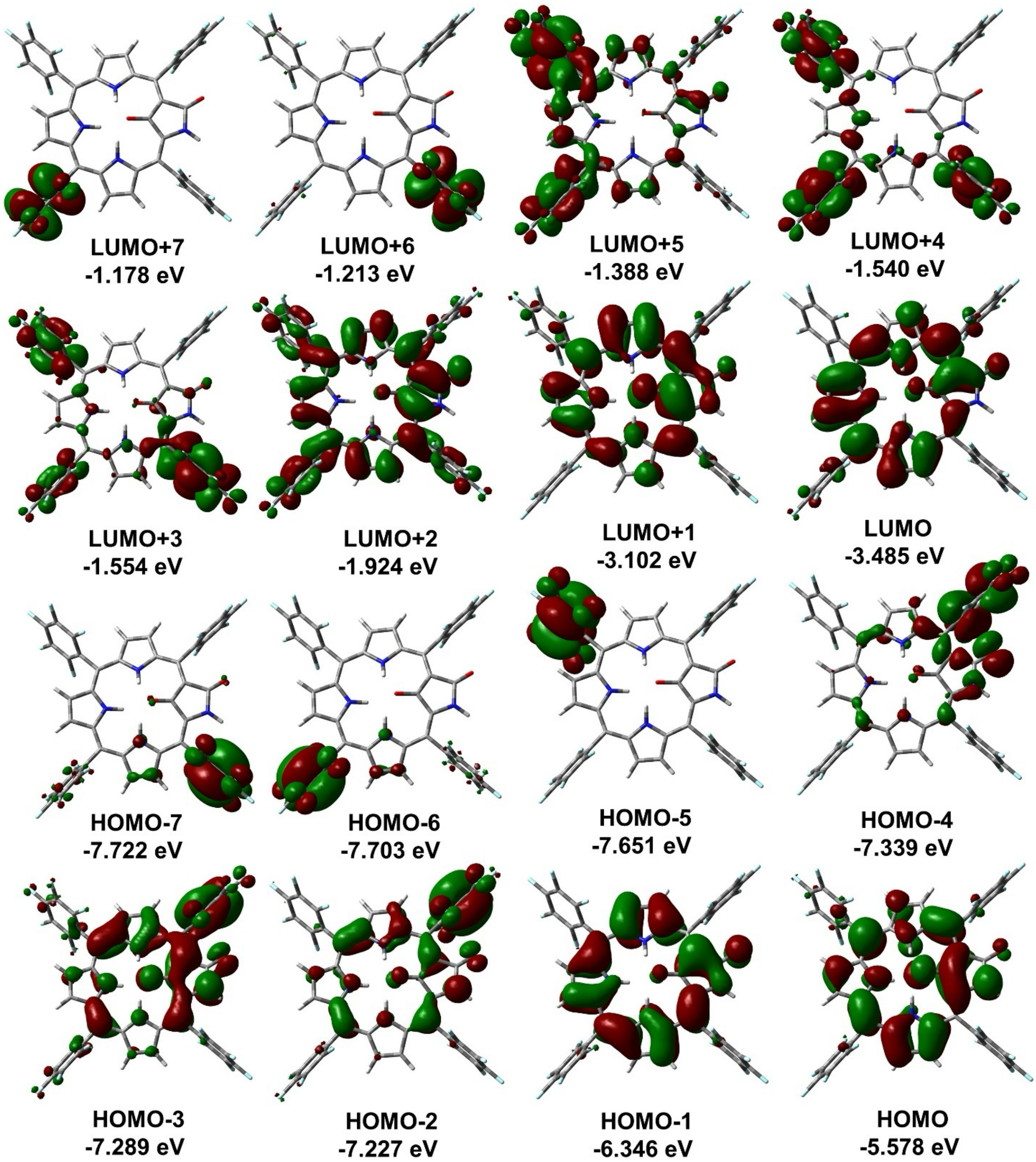

Figure S22 Selected frontier MOs, along with their orbital energies of the 3,21- dioxo-*H_3*PFNCP,**4**

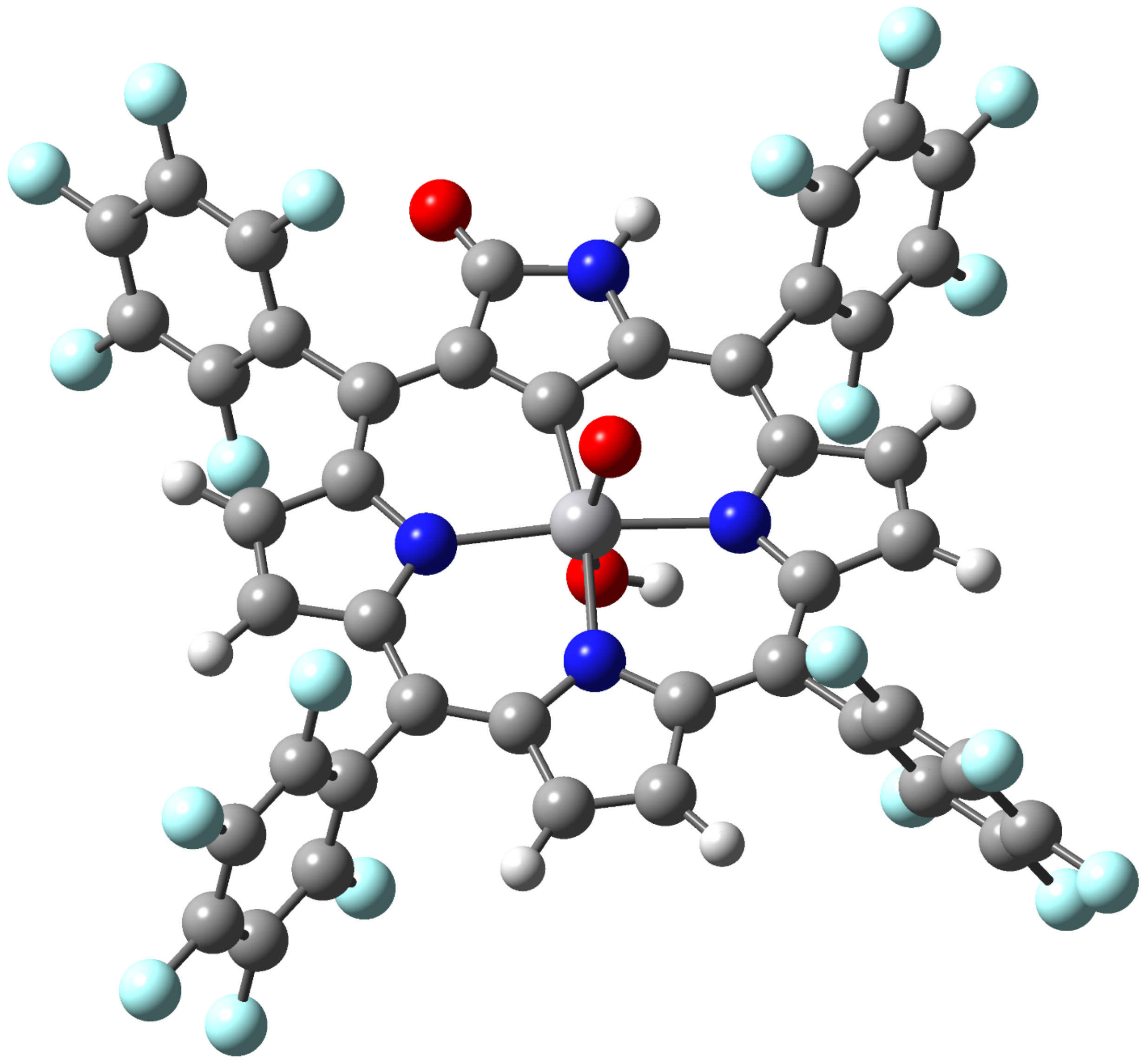

Figure S23 DFT- optimized geometry of V(=O)(OH)(3-oxo-PFNCP),**5** using the 6-311G (d, p) basis set.

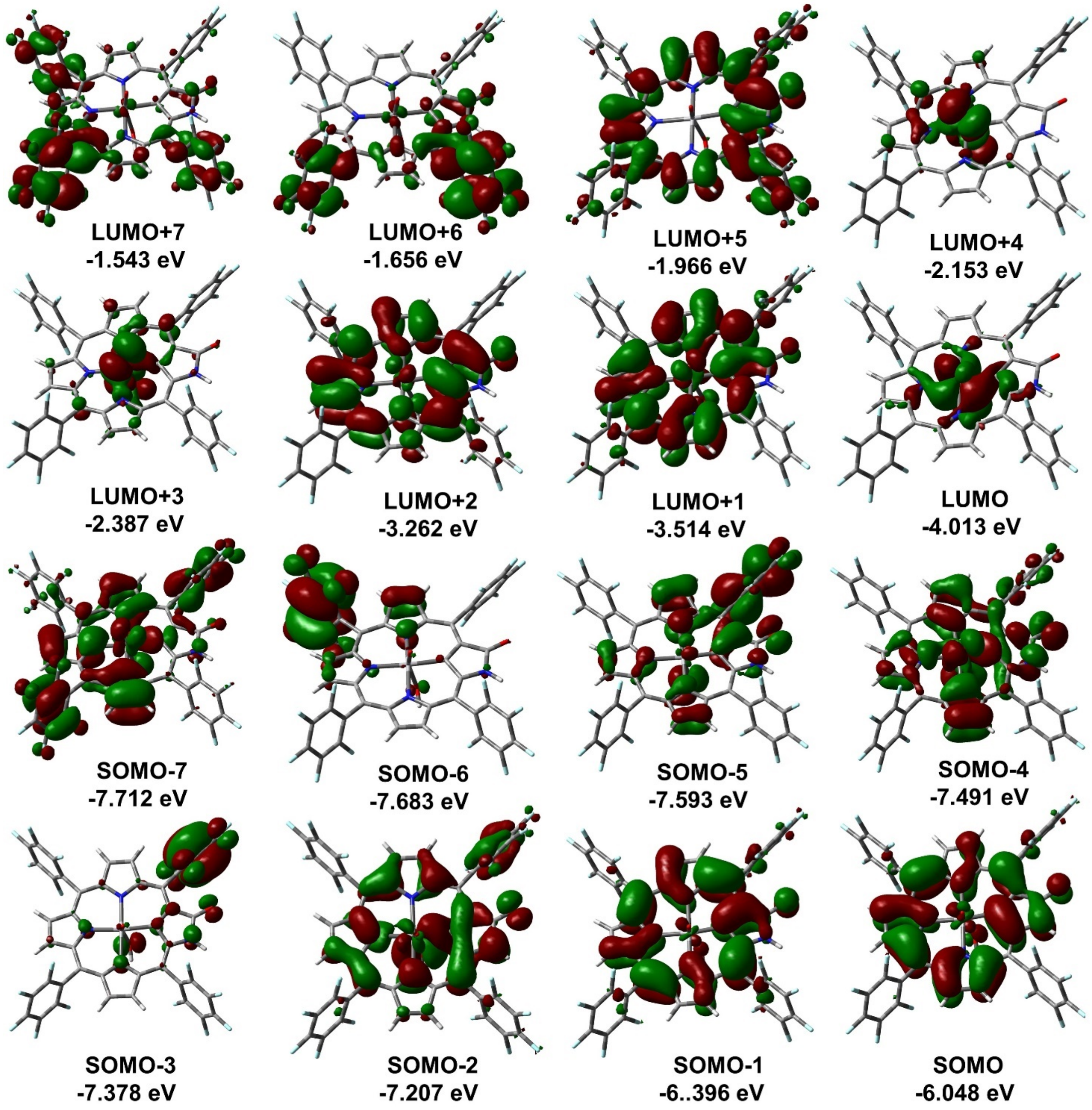

Figure S24 Selected frontier MOs, along with their orbital energies of the V(=O)(OH)(3-oxo-PFNCP),**5**

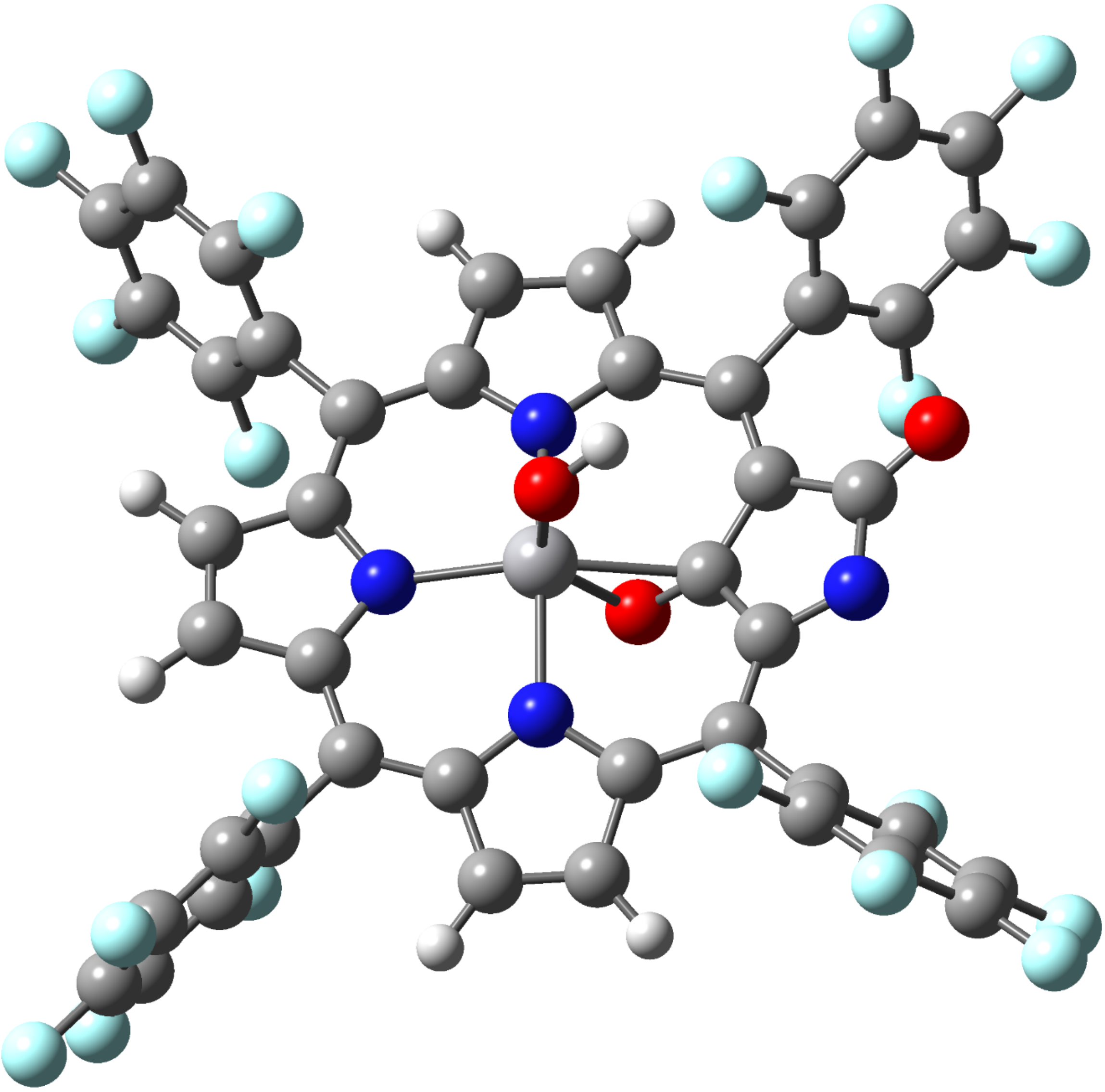

Figure S25 DFT- optimized geometry of $[n\text{-}BnN_4]^+$ **[**V(OH)(3,21-dioxo-PFNCP)$]^-$, **6** using the 6-311G (d, p) basis set.

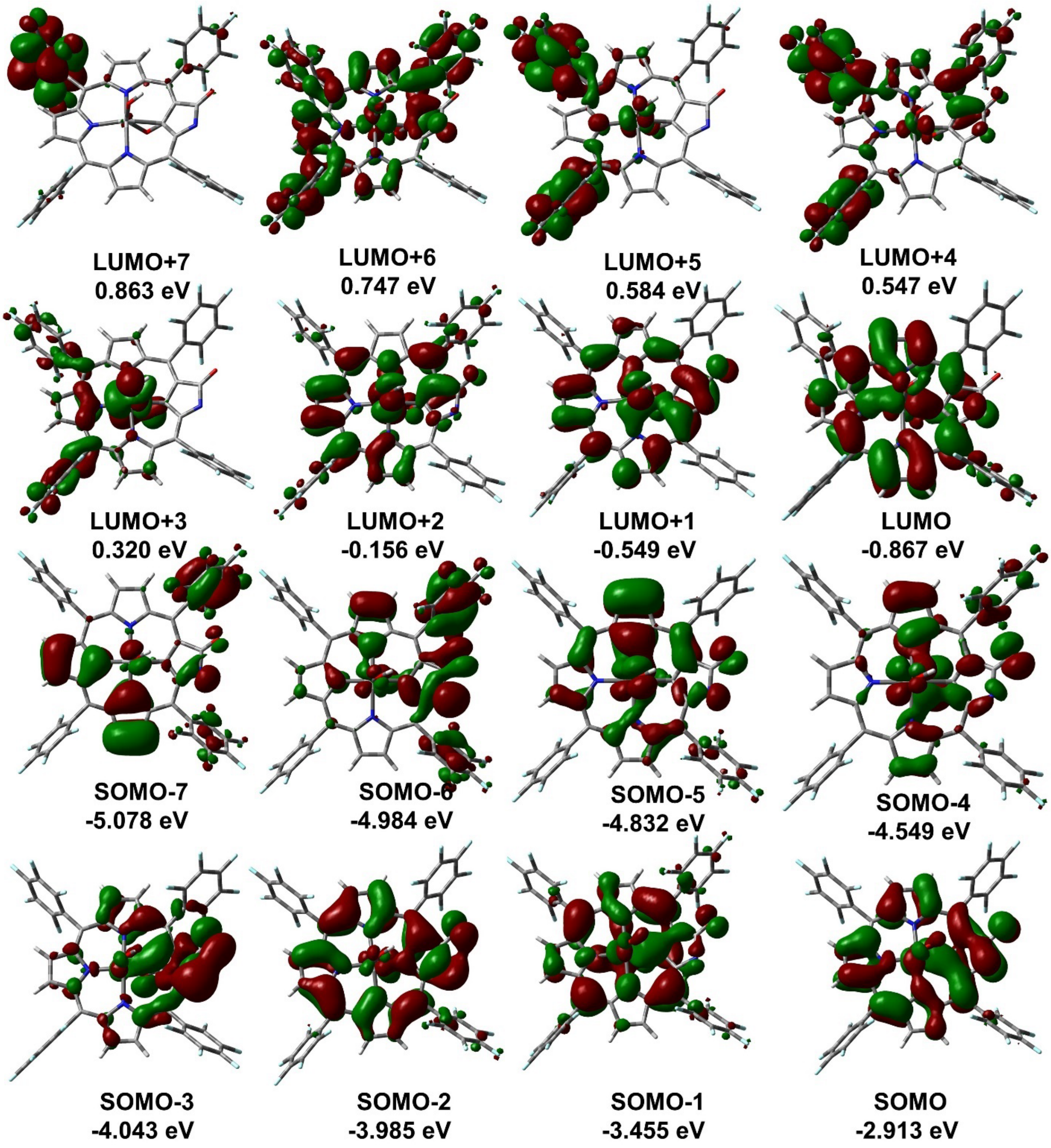

Figure S26 Selected frontier MOs, along with their orbital energies of the $[n\text{-}BnN_4]^+$ $[V(OH)(3,21\text{-dioxo-PFNCP})]^-$, **6**

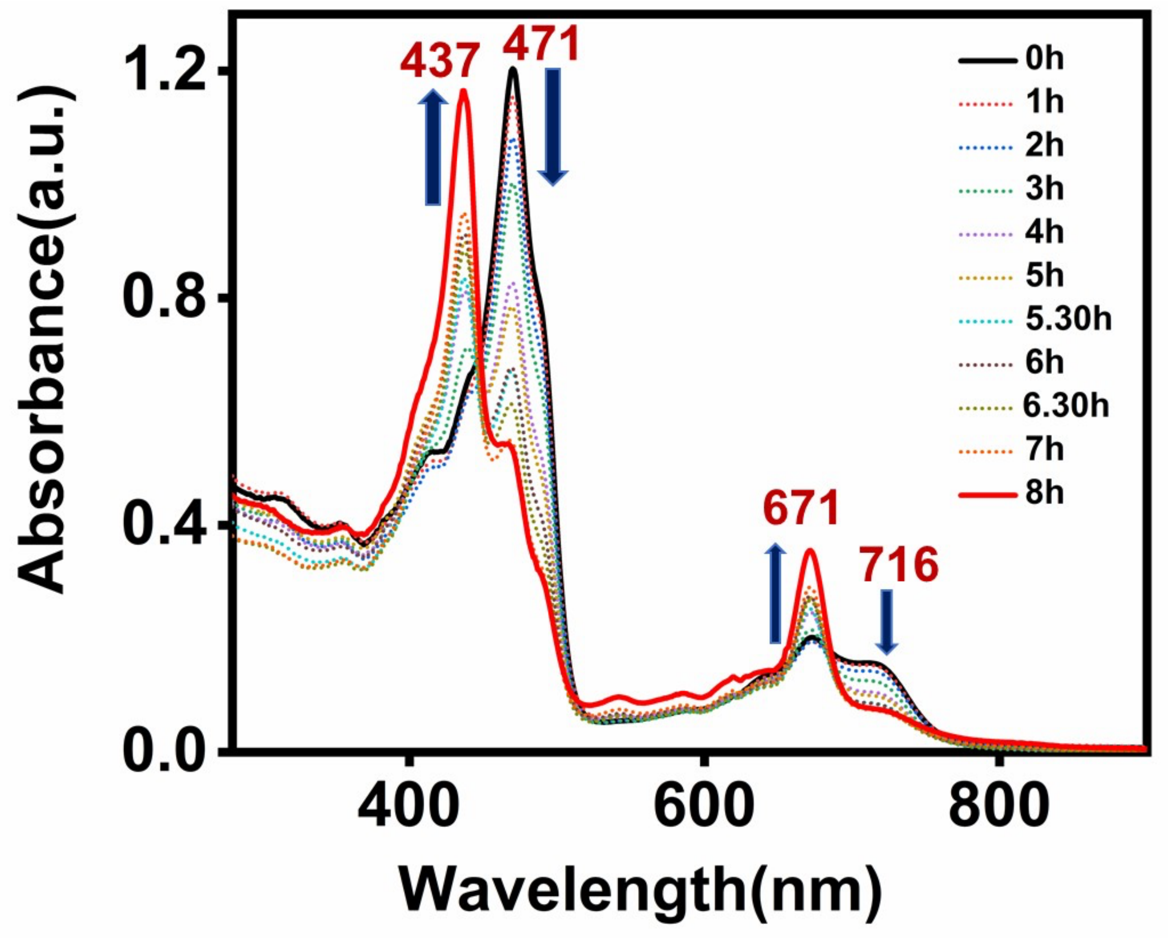

Figure S27 Electronic Absorption Spectra of the reaction mixture of cyclohexene oxidation by compound **6**

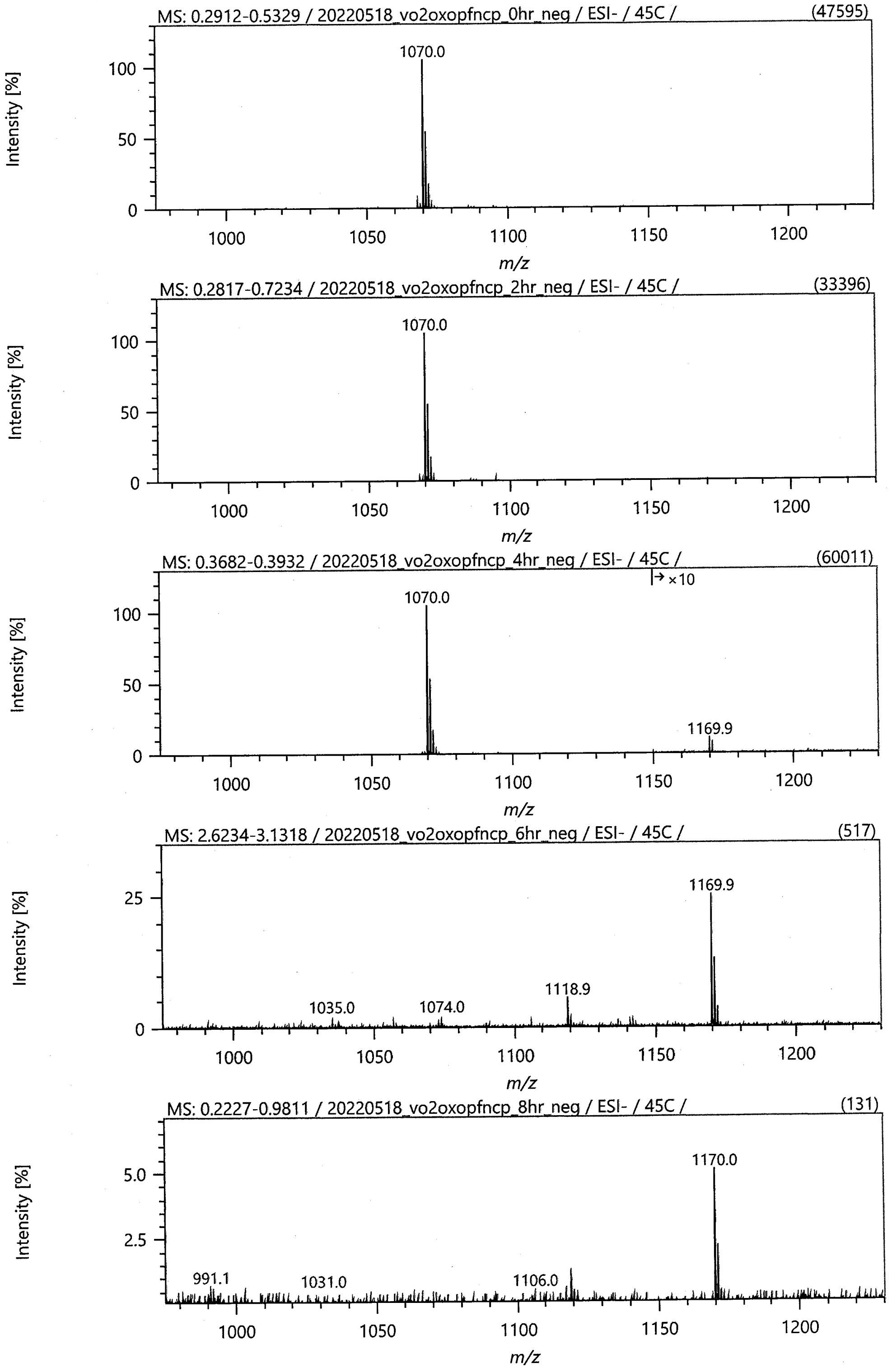

Figure S28a HR-ESI- MS spectrum the reaction mixture of cyclohexene oxidation **(0-8h)** of compound **6**

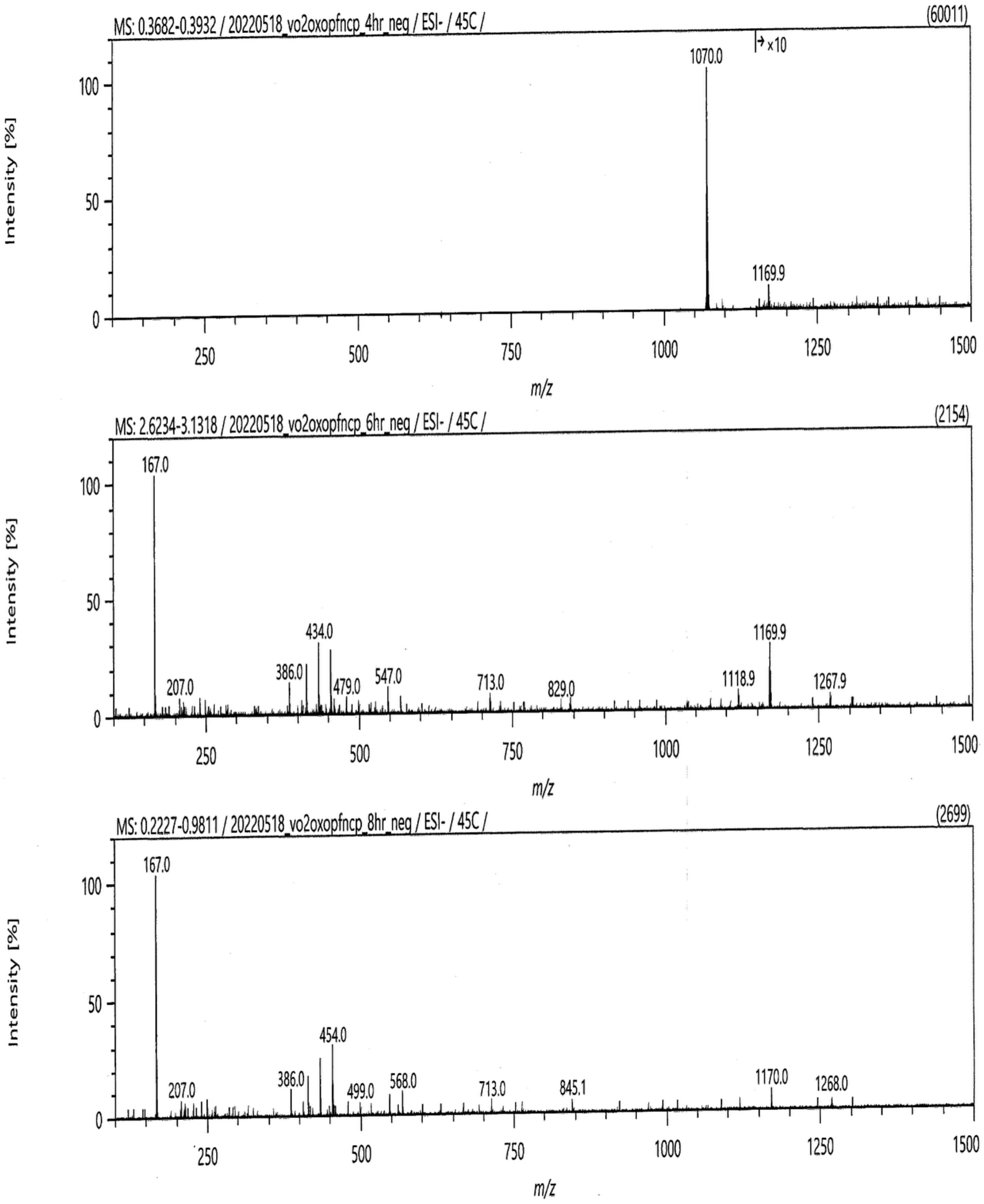

Figure S28b LR-ESI- MS spectrum the reaction mixture of cyclohexene oxidation **(4-8h)** of compound **6**

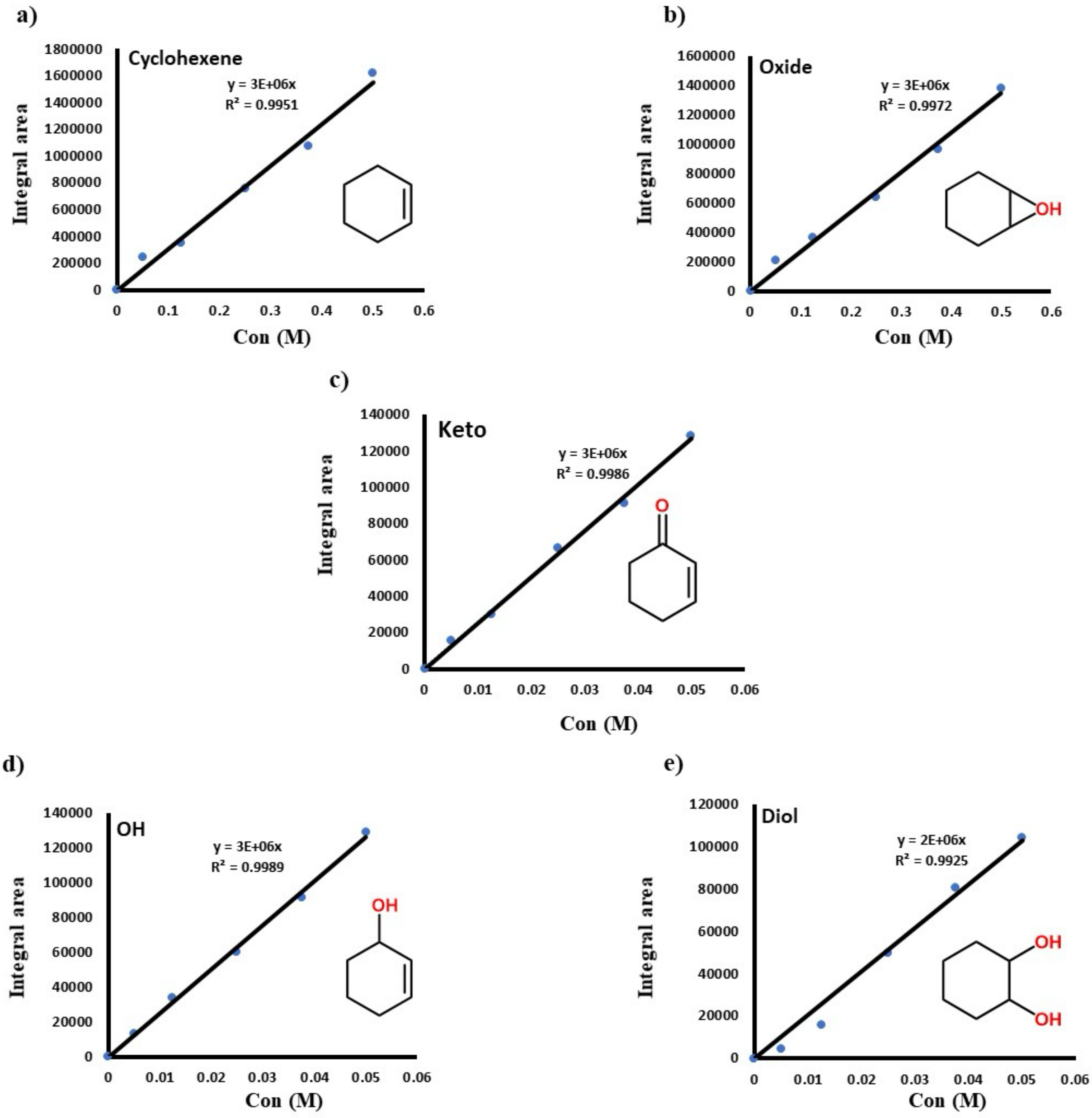

Figure S29 Calibration curves were generated for cyclohexene (a), cyclohexene oxide (b), 2-cyclohexen-1-ol (c), and cyclohexen-1-one (d) using hexane as the solvent. Respectively, calibration curves for cyclohexane-1,2-diol (e) were established using acetonitrile.

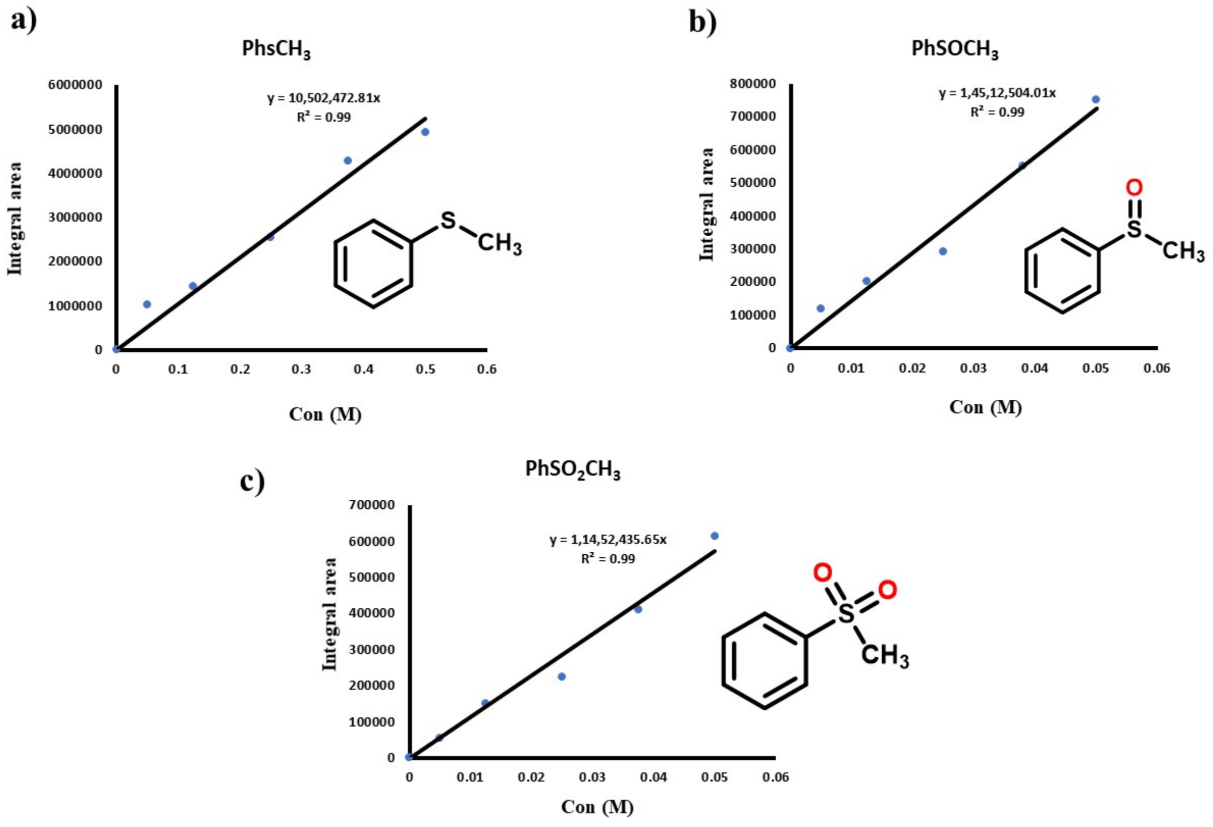

Figure S30 Calibration curves were generated for $PhSCH_3$(a), $PhSOCH_3$ (b), and $PhSO_2CH_3$ (c) using methanol as the solvent.

Table S1 FT-IR Stretching Frequency of all the compounds.

Vibrational modes Stretching Frequency	Compound 3		Compound 4		Compound 5		Compound 6	
	Exp. (cm^{-1})	DFT (cm^{-1})	Exp. (cm^{-1})	DFT (cm^{-1})	Exp. (cm^{-1})	DFT (cm^{-1})	Exp. (cm^{-1})	DFT (cm^{-1})
Aromatic C=O Stretching Frequency	1706	1790	1703	1642 (Inner)<br>1791 (Outer)	1733	1798	1695	1734
Aromatic C-O (V-O-C) Stretching Frequency	--------	--------	--------	--------	--------	--------	1324	1376
V=O Stretching Frequency	--------	--------	--------	--------	951	1023	--------	--------
V-OH Stretching Frequency	--------	--------	--------	--------	445	525	755	761
V-O (V-O-C) Stretching Frequency	--------	--------	--------	--------	--------	--------	487	520
OH Stretching Frequency	--------	--------	--------	--------	3441	3865	(3331-3302)	3843
C-N single bond (O=C-N) Stretching Frequency	1084	1138	1083	1128	1081	1127	1069	1133
NH Stretching Frequency	3373	3591<br>3617 (Inner)<br>3655 (Outer)	3290	3068<br>3132<br>3650 (Inner)<br>3641 (Outer)	3290	3635 (Outer)	-------	--------
Aromatic Pyrrolic CH Stretching Frequency	2934	(3237-3265)	2934	(3245-3265)		(3241-3266)	(2874-2963)	(3229-3265)

Table S2 UV−vis. data of all the compounds.

Compound	UV−vis. Data [a]<br>λ_{max} / nm (ε / 10^5 $M^{-1}cm^{-1}$)
**3**	319(21743), 433(170942), 528(12966), 573(7855), 630(6210), 686(4064)
**4**	353(40691), 450(57727), 480(54472), 585(4001), 650(4739), 790(7992)
**5**	297(25837), 357(25577), 436(145610), 542(7556), 584(6352), 617(10305), 672(47991)
**6**	315(24389), 352(21981), 414(27568), 471(62632), 488(44311), 674(9714), 716(8731),

[a]In CH_3CN.

Table S3 Experimental and Theoretical bond distances.

Compound 5	Bond length [Å]	Theoretical/Bond length [Å]	Compound 6	Bond length [Å]	Theoretical//Bond length [Å]
V=O	1.593	1.604	V=O	-	-
V-OH	2.210	1.900	V-OH	1.898	1.789
V-C_1	2.079	2.023	V-C_1	2.146	2.023
V-N_1	2.085	2.081	V-N_1	2.147	2.050
V-N_2	2.115	2.131	V-N_2	1.987	2.119
V-N_3	2.041	2.062	V-N_3	2.092	2.075
C=O	1.233	1.208	C=O	1.208	1.220
N-H	0.950	1.008	N-H	-	-
C-O	-	-	C-O	1.41	1.343
V-O	-	-	V-O	1.752	1.858

Table S4 Catalysis study of cyclohexene with compound **5.**

Entry	Catalyst	Cat. loading (M)	Cyclohexene (M)	H_2O_2 (30%) (M)	Temp. (° C)	Rxn. time (h)	Product %	Cyclohexene Conversion (%)	Product selectivity				TOF h^{-1}
									Oxide	Keto	OH	Diol	
1	5	0.002	0.125	0.2	45	0	0	16	0	0	0	0	0
2	5	0.002	0.125	0.2	45	3	0	57	0	0	0	0	0
3	5	0.002	0.125	0.2	45	6	4.3	57	0	61	39	0	0.49
4	5	0.002	0.125	0.2	45	9	14.5	63	5	42	38	15	1.00
5	5	0.002	0.125	0.2	45	12	17.1	67	10	38	37	15	0.88
6	5	0.002	0.125	0.2	45	15	19.2	68	11	38	35	16	0.80
7	5	0.002	0.125	0.2	45	18	25.2	79	11	38	33	18	0.87
8	5	0.002	0.125	0.2	45	21	25.2	84	7	39	30	24	0.74
**9**	**5**	**0.002**	**0.125**	**0.2**	**45**	**24**	**26.0**	**84**	**5**	**39**	**28**	**28**	**0.67**

Table S5 Catalysis study of cyclohexene with compound **6.**

Entry	Catalyst	Cat. loading (M)	Cyclohexene (M)	H_2O_2 (30%) (M)	Temp. (°C)	Rxn. time (h)	Product %	Cyclohexene Conversion (%)	Product selectivity				TOF h^{-1}
									Oxide	Keto	OH	Diol	
1	6	0.002	0.125	0.2	45	0	0	11	0	0	0	0	0
2	6	0.002	0.125	0.2	45	3	0	21	0	0	0	0	0
3	6	0.002	0.125	0.2	45	6	17	40	7	46	36	12	1.79
4	6	0.002	0.125	0.2	45	9	27	68	18	36	35	11	1.91
5	6	0.002	0.125	0.2	45	12	36	69	18	34	34	14	1.91
6	6	0.002	0.125	0.2	45	15	37	74	17	34	33	16	1.54
7	6	0.002	0.125	0.2	45	18	34	76	14	34	33	19	1.29
8	6	0.002	0.125	0.2	45	21	36	77	13	35	33	19	1.09
**9**	**6**	**0.002**	**0.125**	**0.2**	**45**	**24**	**38**	**79**	**12**	**35**	**33**	**20**	**1.09**

Table S6: Catalysis study of Thioanisole with **H_2O_2.**

Entry	Cat. loading (M)	Thioanisole (M)	H_2O_2 (30%) (M)	Rxn. time (h)	Product %	Thioanisole Conversion (%)	Product selectivity	
							$PhSOCH_3$	PhS_2OCH_3
1	-	0.125	0.65	4	11	11	100	-
2	-	0.125	0.65	8	21	21	100	-
3	-	0.125	0.65	12	21	21	100	-
4	-	0.125	0.65	16	25	25	100	-
5	-	0.125	0.65	20	27	27	100	-

Table S7 Catalysis study of Thioanisole with compound **5**

Entry	Catalyst	Cat. loading (M)	Thioanisole (M)	H_2O_2 (30%) (M)	Rxn. time (h)	Product %	Thioanisole Conversion (%)	Product selectivity		TOF h^{-1}
								$PhSO_2CH_3$	$PhSOCH_3$	
1	5	0.0006	0.125	0.65	4	36	25	58	42	18.9
2	5	0.0006	0.125	0.65	8	41	35	51	49	10.7
**3**	**5**	**0.0006**	**0.125**	**0.65**	**12**	**69**	**95**	**36**	**64**	**11.9**
4	5	0.0006	0.125	0.65	16	39	99	61	39	5.13
5	5	0.0006	0.125	0.65	20	38	99	67	33	3.98

Table S8: Catalysis study of Thioanisole with compound **6.**

Entry	Catalyst	Cat. loading (M)	Thioanisole (M)	H_2O_2 (30%) (M)	Rxn. time (h)	Product %	Thioanisole Conversion (%)	Product selectivity		TOF h^{-1}
								$PhSO_2CH_3$	$PhSOCH_3$	
1	6	0.0006	0.125	0.65	4	36	25	67	33	18.3
2	6	0.0006	0.125	0.65	8	50	35	67	33	13.0
3	6	0.0006	0.125	0.65	12	60	95	55	45	10.9
4	6	0.0006	0.125	0.65	16	84	99	80	20	11.0
**5**	**6**	**0.0006**	**0.125**	**0.65**	**20**	**87**	**99**	**96**	**4**	**8.99**

Optimized Cartesian Co-ordinates of 3-oxo-*H2*PFNCP, 3

Cartesian coordinates of the optimized structure (in Å). 3-oxo-H_2PFNCP, **3** was optimized at the B3LYP level of theory and 6-311G (d, p) basis set.

3-oxo-*H2*PFNCP, 3

(E = -3974.58 hartrees)

F	2.18910	4.56810	2.12730
F	3.89780	6.64940	2.26550
F	5.65570	7.11130	0.23990
F	5.69210	5.47770	-1.93630
F	3.98040	3.39770	-2.09610
F	3.25950	-3.90090	2.04840
F	5.42240	-5.51300	2.12540
F	7.15430	-5.57650	0.02390
F	6.70450	-4.01940	-2.16380
F	4.54430	-2.39920	-2.25040
F	-3.78290	-3.50890	-2.21260
F	-5.51180	-5.58470	-2.16160
F	-5.83970	-7.03050	0.12140
F	-4.42550	-6.39170	2.35850
F	-2.69210	-4.31930	2.31780
F	-4.47470	2.48380	-2.29560
F	-6.62880	4.11270	-2.33770
F	-7.29400	5.55240	-0.12520
F	-5.78900	5.35730	2.13530
F	-3.63160	3.73270	2.18860
O	5.21060	-0.69950	0.64220

N	-0.33410	2.16350	-0.03620
N	0.16430	-2.14610	-0.08200
C	-1.55060	2.79740	-0.13350
C	-1.26870	4.17800	-0.35340
H	-2.01140	4.94660	-0.49250
C	0.09450	4.33620	-0.37160
H	0.63700	5.25270	-0.53690
C	0.70010	3.05990	-0.16460
C	2.07760	2.76630	-0.08700
C	2.59700	1.46870	-0.04760
C	2.85570	-0.81070	-0.09910
C	2.64620	-2.19940	-0.15690
C	1.37600	-2.79270	-0.20440
C	1.06570	-4.18510	-0.34210
H	1.79920	-4.96140	-0.48540
C	-0.29190	-4.33670	-0.27900
H	-0.84580	-5.25800	-0.35660
C	-0.87610	-3.04160	-0.10280
C	-2.22420	-2.70110	0.02790
C	-2.75520	-1.39440	0.11840
C	-3.00880	0.76960	0.09320
C	-2.79020	2.15720	-0.02300
C	3.02290	3.91510	0.00720
C	3.03470	4.77320	1.11100
C	3.91010	5.84870	1.19760
C	4.81120	6.08460	0.16550
C	4.83030	5.24690	-0.94410
C	3.94360	4.17950	-1.01180
C	3.82320	-3.11540	-0.11430
C	4.08510	-3.92180	0.99220
C	5.19780	-4.75070	1.05030

C	6.08430	-4.78150	-0.01850
C	5.85210	-3.98660	-1.13500
C	4.73440	-3.16660	-1.16870
C	-3.18600	-3.84770	0.05200
C	-3.92420	-4.20260	-1.07710
C	-4.81780	-5.26690	-1.06540
C	-4.98590	-6.00710	0.09920
C	-4.26240	-5.67990	1.24000
C	-3.37510	-4.61080	1.20420
C	-3.98770	3.05410	-0.05080
C	-4.77850	3.17860	-1.19340
C	-5.88990	4.01210	-1.22960
C	-6.23040	4.74930	-0.10130
C	-5.46030	4.64920	1.05150
C	-4.35340	3.80890	1.06390
H	4.58580	1.84840	0.65320
H	-5.26130	0.75170	0.31720
C	1.96460	0.23900	-0.32650
C	-4.33900	0.19890	0.23780
C	-4.18190	-1.14280	0.25530
N	-2.05970	-0.21920	0.04680
N	3.91420	1.18380	0.30400
H	-4.95200	-1.89150	0.35060
C	4.16180	-0.18480	0.32060
H	1.00600	0.16300	-0.80850
H	-0.00520	-1.17030	0.11330
H	-0.28320	1.18470	0.20700

Optimized Cartesian Co-ordinates of 3,21-dioxo-*H3*PFNCP, 4

Cartesian coordinates of the optimized structure (in Å). 3,21-dioxo-*H3*PFNCP, **4** was optimized at the B3LYP level of theory and 6-311G (d, p) basis set.

3,21-dioxo-*H3*PFNCP, 4

(E = -4049.84 hartrees)

F	3.25970	4.27120	-2.31030
F	4.92040	6.35770	-1.86840
F	5.44470	7.16670	0.67590
F	4.30010	5.88030	2.78380
F	2.63680	3.79140	2.35270
F	-3.69540	3.80120	-2.26520
F	-5.88110	5.38130	-2.18690
F	-7.48190	5.38940	0.01440
F	-6.88170	3.80590	2.14590
F	-4.69660	2.22600	2.08590
F	-3.84280	-3.40500	2.13970
F	-5.48750	-5.54080	2.23160
F	-5.60520	-7.26640	0.12900
F	-4.06640	-6.83910	-2.07580
F	-2.42330	-4.70190	-2.18630
F	3.58360	-3.13340	2.27660
F	5.82030	-4.61980	2.58380
F	7.35770	-5.23370	0.42040
F	6.64180	-4.35870	-2.05670
F	4.40930	-2.86380	-2.37060
O	0.75080	0.12860	-1.18210
O	5.26960	-0.49390	0.10870

N	3.90150	1.32240	-0.23780
H	4.57140	1.99450	0.10210
C	4.17540	-0.04330	-0.15230
N	-0.43900	2.21860	-0.30600
H	-0.22570	1.32380	-0.77720
N	-2.15340	-0.29520	-0.07880
H	-1.22350	-0.15030	0.27990
N	0.23120	-2.19830	-0.23360
H	0.17340	-1.28050	-0.71020
C	2.53840	1.57620	-0.41010
C	1.91840	0.27370	-0.69960
C	2.87800	-0.74060	-0.40530
C	1.96720	2.82200	-0.24190
C	0.56950	3.13200	-0.15710
C	-0.04180	4.35980	0.19610
H	0.47670	5.27900	0.41560
C	-1.41170	4.15240	0.24930
H	-2.16170	4.87880	0.51770
C	-1.66160	2.79430	-0.07080
C	-3.10140	0.72510	-0.19890
C	-4.35150	0.06020	-0.42290
H	-5.28480	0.56680	-0.60760
C	-4.14640	-1.28650	-0.41240
H	-4.88480	-2.05190	-0.58540
C	-2.75280	-1.54310	-0.17820
C	-2.15650	-2.80670	-0.07920
C	-0.76890	-3.09810	-0.02480
C	-0.13880	-4.35540	0.24160
H	-0.65570	-5.27040	0.48130
C	1.22130	-4.17690	0.16020
H	1.97890	-4.92550	0.32620

C	1.47230	-2.80520	-0.14360
C	2.70020	-2.12850	-0.24040
C	2.89670	3.96980	0.00780
C	3.50190	4.64820	-1.05110
C	4.35760	5.72280	-0.83820
C	4.62580	6.13830	0.46120
C	4.04000	5.48040	1.53690
C	3.18820	4.40790	1.30010
C	-2.90160	2.10270	-0.12810
C	-4.12400	2.95980	-0.09330
C	-4.45880	3.78810	-1.16710
C	-5.58160	4.60590	-1.14190
C	-6.40210	4.60950	-0.01960
C	-6.09560	3.79790	1.06630
C	-4.96730	2.98810	1.01960
C	-3.07710	-3.98050	-0.02730
C	-3.88050	-4.23190	1.08760
C	-4.73270	-5.32760	1.15030
C	-4.79250	-6.21130	0.07910
C	-4.00410	-5.99240	-1.04470
C	-3.16280	-4.88750	-1.08650
C	3.92240	-2.97520	-0.06050
C	4.31510	-3.43450	1.19340
C	5.46580	-4.19150	1.36790
C	6.25280	-4.50220	0.26630
C	5.88500	-4.05610	-0.99770
C	4.73280	-3.29800	-1.14590

Optimized Cartesian Co-ordinates of V(=O)(OH)(3-oxo-PFNCP), 5

Cartesian coordinates of the optimized structure (in Å). V(=O)(OH)(3-oxo-PFNCP), **5** was optimized at the B3LYP level of theory and 6-311G (d, p) basis set. The LANL2DZ pseudopotential was used for the Vanadium atom.

V(=O)(OH)(3-oxo-PFNCP), 5

(E = -4195.23 hartrees)

V	0.09040	-0.07970	-0.14390
F	-2.38730	-4.51860	-2.17520
F	-3.37470	-7.03040	-2.04390
F	-3.25460	-8.42240	0.29100
F	-2.14040	-7.29150	2.50140
F	-1.14840	-4.77910	2.38180
F	-4.96330	1.17750	-2.28170
F	-7.48580	2.15110	-2.25810
F	-8.43900	3.40620	-0.03850
F	-6.85480	3.68990	2.15800
F	-4.33240	2.72690	2.14130
F	2.14770	4.49810	2.43810
F	3.12790	7.01620	2.53160
F	3.39370	8.46070	0.23990
F	2.67410	7.37640	-2.15080
F	1.68830	4.85970	-2.25310
F	4.68740	-2.41520	2.13860
F	7.22130	-3.34180	2.00100
F	8.59850	-3.20390	-0.34320
F	7.42800	-2.13350	-2.55480
F	4.89460	-1.20050	-2.42780
O	-0.18340	-0.40130	1.70940

H	0.09730	-1.20550	2.15610
O	0.07000	-0.48340	-1.69640
O	-5.33570	-0.95810	0.08700
N	0.96900	-1.93600	0.05260
H	-3.93570	-3.16910	0.14100
N	-0.74760	1.87910	-0.06690
H	4.24620	3.25130	0.14640
C	2.30010	-2.23440	-0.04840
C	2.43500	-3.67710	-0.12140
H	3.36410	-4.21870	-0.19980
C	1.18000	-4.19540	-0.07940
H	0.90290	-5.23650	-0.12210
C	0.25370	-3.07890	0.01350
C	-1.17730	-3.18550	0.03510
C	-2.07330	-2.12860	0.00530
C	-3.04830	-0.04330	-0.06690
C	-4.13360	-1.06700	0.03220
C	-3.19320	1.36030	-0.08920
C	-2.09360	2.22730	-0.09880
C	-2.21610	3.65990	-0.11180
H	-3.14500	4.20460	-0.15270
C	-0.96140	4.17290	-0.06180
H	-0.67840	5.21250	-0.04400
C	-0.05240	3.05750	-0.03220
C	1.35130	3.21050	0.03490
C	2.27740	2.17210	0.04890
C	3.13010	0.13670	-0.02500
C	4.23320	1.06480	0.04600
H	5.27600	0.79350	0.06850
C	3.30690	-1.27540	-0.07140
C	-1.73830	-4.57000	0.10030

C	-2.31730	-5.17980	-1.01500
C	-2.82950	-6.47130	-0.96220
C	-2.76740	-7.18470	0.22960
C	-2.19720	-6.60550	1.35810
C	-1.69480	-5.31250	1.28210
C	-4.56750	1.94010	-0.08080
C	-5.40780	1.80060	-1.18460
C	-6.70510	2.29060	-1.18380
C	-7.19380	2.93050	-0.05060
C	-6.38330	3.07720	1.06810
C	-5.08570	2.58210	1.04290
C	1.88890	4.60550	0.08940
C	2.26660	5.18830	1.29850
C	2.77290	6.48120	1.36100
C	2.90910	7.22040	0.19150
C	2.54070	6.66530	-1.02880
C	2.03780	5.37040	-1.06700
C	4.71350	-1.78010	-0.14160
C	5.34140	-2.33550	0.97320
C	6.64420	-2.81630	0.91770
C	7.34920	-2.74570	-0.27820
C	6.75000	-2.19750	-1.40680
C	5.44620	-1.72360	-1.32770
C	-1.84050	-0.68270	-0.08270
N	-3.44600	-2.29080	0.06230
N	1.95230	0.84270	-0.01520
C	3.70990	2.31770	0.09290

Optimized Cartesian Co-ordinates of [n-BnN$_4$]$^+$[V(OH)(3,21-dioxo-PFNCP)]$^-$, 6

Cartesian coordinates of the optimized structure (in Å). [n-BnN$_4$]$^+$[V(OH)(3,21-dioxo-PFNCP)]$^-$, **6** was optimized at the B3LYP level of theory and 6-311G (d, p) basis set. The LANL2DZ pseudopotential was used for the Vanadium atom.

[n-BnN$_4$]$^+$ [V(OH)(3,21-dioxo-PFNCP)]$^-$, 6

(E = -4194.82 hartrees)

V	-0.08940	-0.02940	0.16200
F	-4.72550	-2.19390	-2.27500
F	-6.98530	-3.66540	-2.25030
F	-7.70280	-5.03280	-0.00620
F	-6.13950	-4.91490	2.22180
F	-3.88120	-3.44210	2.21520
F	-2.36440	4.48270	2.26740
F	-3.88010	6.71480	2.31040
F	-5.17990	7.52760	0.05860
F	-4.95180	6.08980	-2.24370
F	-3.43730	3.85720	-2.30130
O	0.29440	-0.00320	1.90800
H	1.15360	0.17030	2.30660
N	0.31290	2.03240	-0.11930
H	-5.32310	-0.32960	0.27990
N	-0.51540	-2.04850	-0.05880
C	-1.60940	-4.05590	-0.30950
H	-2.42080	-4.76050	-0.40540
C	-1.75670	-2.62800	-0.12250
C	-2.96840	-1.94950	-0.03960

C	-3.11160	-0.54700	0.03160
C	-2.67440	1.62940	0.01870
C	-4.08710	1.47920	0.16760
H	-4.79550	2.28580	0.27250
C	-2.00260	2.85790	-0.04560
C	-0.62080	3.04110	-0.14960
C	0.04030	4.30480	-0.33990
H	-0.44620	5.26470	-0.41590
C	-4.21980	-2.76460	-0.03080
C	-5.04680	-2.84800	-1.15270
C	-6.21440	-3.60180	-1.15570
C	-6.58150	-4.30190	-0.01370
C	-5.78160	-4.24180	1.11960
C	-4.61970	-3.47840	1.10020
C	-2.84780	4.09210	-0.01860
C	-2.98550	4.85310	1.14230
C	-3.76320	6.00490	1.17990
C	-4.42810	6.42060	0.03380
C	-4.31150	5.68480	-1.13820
C	-3.52800	4.53670	-1.15240
F	1.77250	-4.73440	2.01020
F	3.38740	-6.88000	2.19690
F	5.16620	-7.43630	0.20650
F	5.30580	-5.82410	-1.98570
F	3.67990	-3.68520	-2.19960
F	4.77400	2.20750	-2.29420
F	7.01970	3.67650	-2.01870
F	7.49430	5.02880	0.29850
F	5.69600	4.90410	2.34440
F	3.44710	3.43650	2.08200
O	0.88900	-0.19780	-1.40900

O	5.19530	0.22560	0.53580
C	-0.28210	-4.31720	-0.36560
H	0.19310	-5.27320	-0.51960
C	0.41700	-3.05590	-0.22020
C	1.80470	-2.92080	-0.21250
C	2.48870	-1.67410	-0.17290
C	2.91760	0.57680	-0.26650
C	4.11220	-0.22980	0.20390
C	2.79370	1.95140	-0.26330
C	1.53980	2.62090	-0.29090
C	1.37430	4.04350	-0.42560
H	2.17360	4.75010	-0.58500
C	2.65640	-4.13580	-0.11080
C	2.61920	-4.98010	0.99970
C	3.45120	-6.08700	1.11570
C	4.36200	-6.36870	0.10780
C	4.43190	-5.54390	-1.00750
C	3.58900	-4.44560	-1.10360
C	4.02330	2.78760	-0.12410
C	4.96990	2.86440	-1.14450
C	6.13180	3.61140	-1.01510
C	6.37690	4.30100	0.16490
C	5.45740	4.24060	1.20230
C	4.30030	3.48880	1.04760
C	1.91480	-0.38350	-0.56180
N	3.74530	-1.57110	0.26010
C	-4.35670	0.13890	0.17980
N	-2.08780	0.37280	-0.05980
